# Synthesis, characterization and sorption studies of aromatic compounds by hydrogels of chitosan blended with β-cyclodextrin- and PVA-functionalized pectin†

**DOI:** 10.1039/c8ra02332h

**Published:** 2018-04-18

**Authors:** Cesar M. C. Filho, Pedro V. A. Bueno, Alan F. Y. Matsushita, Adley F. Rubira, Edvani C. Muniz, Luísa Durães, Dina M. B. Murtinho, Artur J. M. Valente

**Affiliations:** CQC, Department of Chemistry, University of Coimbra 3004-535 Coimbra Portugal avalente@ci.uc.pt +351 239852080; Grupo de Materiais Poliméricos e Compósitos (GMPC) – Departamento de Química, Universidade Estadual de Maringá, UEM 87020-900 Maringá PR Brazil; Post-graduate Program on Materials Science & Engineering, Federal University of Technology, Paraná (UTFPR-LD) 86036-370 Londrina PR Brazil; CIEPQPF, Department of Chemical Engineering, University of Coimbra Rua Sílvio Lima 3030-790 Coimbra Portugal

## Abstract

Petroleum comprises the monoaromatic and polycyclic aromatic hydrocarbons, which exhibit acute toxicity towards living animals. Consequently, their removal from natural environment is a priority challenge. On the other hand, biomaterials are increasingly being used as adsorbents. Pectin and chitosan are well-known polysaccharides able to form coacervate hydrogels. Aiming an increase of sorption ability by hydrophobic compounds, pectin was also functionalized with two amphiphilic compounds: β-cyclodextrin (β-CD) and poly(vinyl alcohol) (PVA). Both the modified pectin and the hydrogels were evaluated using nuclear magnetic resonance (NMR), infrared spectroscopy (FTIR), and scanning electron microscopy (SEM). The hydrogels were further characterized in terms of thermogravimetric analysis (TGA) and swelling kinetics. The interaction between the hydrogel and mix solutions containing six different aromatic compounds (BTXs and the following PAHs: pyrene, benzo(b)fluoranthene and benzo(a)pyrene) has been evaluated through sorption isotherms and kinetics. The mechanism of sorption interaction and the selectivity of the adsorbents towards different aromatic compounds were discussed. The results clearly show that the presence of β-CD and PVA into gel leads to an increase in the removal efficiency of both, BTXs and PAHs. The gels were subjected to two sorption/desorption cycles to have an assessment of the capability of adsorbents for re-use. Finally, the sorption quantification of those six aromatic compounds from a real gasoline sample onto gels has been tested.

## Abstract 2

Complex coacervation of chitosan with β-cyclodextrin- and poly(vinyl alcohol)-functionalized pectin: ability for simultaneous removal of six different aromatic compounds.

## Graphical abstract

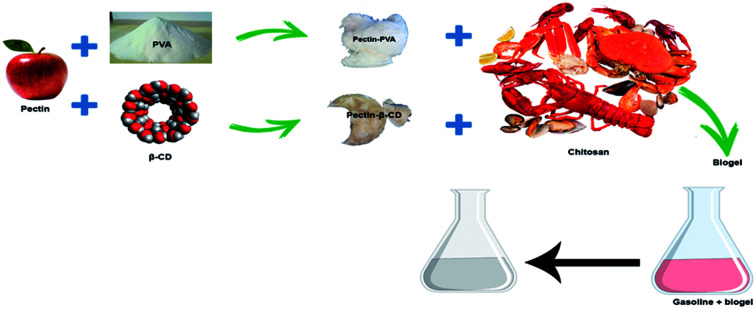

## 1. Introduction

Polycyclic aromatic hydrocarbons (PAHs) (*e.g.*, pyrene (pyr), benzo(b)fluoranthene (B(b)F) and benzo(a)pyrene (B(a)P) and the monoaromatic hydrocarbons (MAHs) (*e.g.*, benzene, toluene and xylene isomers (BTXs)), are carcinogenic, teratogenic and mutagenic agents in humans^1,2^ and environmental priority pollutants.^3,4^

These hydrocarbons are naturally present in crude petroleum and fossil fuels products, such as gasoline and diesel fuel.^5,6^ BTXs constitute a significant percentage of petroleum products; for example, the commercial gasoline contains between 18 to 25% (w/w) of those compounds.^5^ On the other hand, the overall concentration of pyrene, B(b)F and B(a)P in standard gasoline only corresponds to 0.002% (w/w).^7^

In the costal zones, PAHs and MAHs present in sewers, industrial effluents and those generated by forest fires can pollute groundwater and drinking water reservoirs.^6^ At sea, the aromatic hydrocarbons severely pollute sea water, predominantly through oil seeps and spills, and water discharged from offshore oil installations.^6^ Table S1,† shows a resume of concentration ranges of these compounds in different real samples.

Nowadays many materials have been used in the remediation of water contaminated with MAHs and PAHs, such as activated carbon, zeolites, microorganisms, among others.^8–12^ However, these methods show some drawbacks, including the removal of trace amounts of those hydrocarbons and the cost related to pre- and post-treatments is considerable.^8^

The use of low cost and environmentally friendly sorbents in the remediation of contaminated environments, especially those consisting of natural polymers, have been receiving significant interest in the last decades.^11,13^

Chitosan (CS) is a polycationic polysaccharide found naturally in the shellfish exoskeleton and crustaceous. Chitosan is the most commonly used cationic biopolymer (positively charged at pH < 6.5).^14^ The CS is non-toxic, biocompatible and is able to form films and therefore it has found many applications in food industry, cosmetic fabrication, among others.^15^ CS has amine and hydroxyl groups in its structure, susceptible of modification, which makes this biopolymer more chemically versatile than others.^16,17^ Recently, numerous papers have been published making the use of CS as adsorbent for a wide range of environmental contaminants, such as organic compounds, mainly because the high content of the referred amino and hydroxyl groups.^18^

Pectin (Pec) is an anionic heteropolysaccharide, based on α-(1-4) linked d-galacturonic acid, present on the cell walls of dicots.^19^ Pectin has been used to remove pollutants (*e.g*., heavy metals and dyes) through the functionalization of carboxylic groups.^11,20^ Furthermore, it has been shown that the chemical modification of the pectin structure can contribute to the improvement of its properties through the increase of swelling degree which reflects in important changes on release and adsorption of solutes.^21^ These modifications, namely transesterification reactions,^22^ allow to evaluate the ability of β-cyclodextrin (β-CD) and poly(vinyl alcohol) (PVA) to improve the pollutant removal/solubilisation.

β-CD is a cyclic oligosaccharide composed by seven glucose units, formed through α-1,4-glucosidic linkages. β-CD has a hydrophobic internal cavity and hydrophilic outer surface. Due to their structure, cyclodextrins readily form inclusion host-guest complexes, through covalent interactions, with a broad range of organic and inorganic guest molecules.^23^ Among the organic compounds that are able to form complexes with β-CD, we may refer BTXs and PAHs in such a way that the use of β-CDs can efficiently contributes for the remediation of contaminated environments.^24,25^

Poly(vinyl alcohol) (PVA) is a water-soluble, non-toxic, non-carcinogenic and easily processed polymer. PVA has also the ability to form physical and chemically-crosslinked hydrogels that exhibit high degree of swelling in water, and a rubbery and elastic nature.^26^ Recently, it has been found that PVA can adopt a myriad of structures, making the PVA to behave as an amphiphilic polymer.^27^ The modification of different substrates with PVA has caused improvements in removal efficiency of BTXs.^28^ This ability has been justified by the interaction between the hydroxyl groups and the π-benzene electron clouds and, in this way, the selectivity and solubility of benzene are improved.^28^

Here we report the synthesis and characterization of blend gels of chitosan and pectin (either in the native state or modified with β-CD or PVA) for the removal of aromatic hydrocarbons. The gels were formed through electrostatic interactions between positively and negatively charged groups of CS and Pec, respectively,^15,29^ by using an oil-in-water (o/w) emulsion.^30^ Both modified pectin and the obtained hydrogels have been evaluated using several techniques, such as thermogravimetric analysis (TGA), Fourier-transform infrared spectroscopy (FTIR) and scanning electron microscopy (SEM). Besides, the swelling properties of the obtained hydrogels were determined gravimetrically and the performance of the hydrogels to the simultaneous sorption of the BTXs and selected PAHs (pyrene, B(a)F and B(a)P) has been also assessed. Finally, the capacity of those hydrogels to BTXs and PAHs removal, from a real gasoline sample, has been measured and discussed.

## 2. Experimental section

### 2.1. Materials

β-cyclodextrin (>98%), pectin from apple (*M*_V_: 9000; 73.9% esterification), sulfuric acid (96.5%), poly(vinyl alcohol) (*M*_w_*ca.* 13 000; 98.0–98.8 mol% hydrolysis), dimethylformamide (DMF) (99%), acetone (99.7%) and dialysis tubing cellulose membrane (molecular weight cut-off: 14 000 Da MWCO) were obtained from Sigma-Aldrich (Germany). Chitosan with acetylation degree of 15 mol% (*M*_wA_: 87 × 10^3^ g mol^−1^) was purchased from Golden-Shell Biochemical (China). Benzyl alcohol (99%) was obtained from Merck KGaA (Germany).

Benzene (ben) (>99.7%) and xylene (xyl) (mixture of isomers >97%) were purchased from Merck KGaA (Germany) and toluene (tol) (>99.8%) was obtained from Lab-Scan (Poland). Pyrene (pyr) (GC grade > 97%), benzo(b)fluoranthene (B(b)F) (HPLC grade > 98%) and benzo(a)pyrene (B(a)P) (>96%) were purchased from Sigma-Aldrich (Germany).

Acetonitrile and methanol, HPLC grade, were purchased from Fisher Scientific (UK) and Sigma-Aldrich (Germany), respectively. Deionized water was obtained using a Millipore© system. All reagents were used without further purification.

### 2.2. Procedure for hydrocarbon quantification

The quantification of BTXs (benzene, toluene and xylenes) and some PAHs (pyrene, benzo(b)fluoranthene and benzo(a)pyrene) was performed in a VWR-Hitachi LaChrom Elite HPLC system (Hitachi, Japan), equipped with a degasser, auto sampler, column oven and diode array detector (DAD), according to the previously optimized method.^31^

An analytical column (0.25 m × 4.6 mm, 5 μm film) Purospher® Star RP-18 end capped (Merck-Millipore, Germany) was used. The data acquisition and processing were done using EZChrom Elite software (Agilent, USA). Hydrocarbons solutions were daily prepared and calibration curves were obtained for each of the hydrocarbons.

Briefly, the following methodology has been used: aqueous solutions of PAHs and BTX were analyzed in HPLC-DAD system, in gradient mode, using a ternary mixture as the mobile phase (methanol, acetonitrile and ultrapure water, 20 : 50 : 30%), by direct injection of each sample (20 μL) with a flow rate of 1.5 mL min^−1^. The detection of BTXs and PAHs were analytically determined at the following wavelengths: *λ*(ben) = 207 nm, *λ*(tol and xyl) = 211 nm, *λ*(pyr) = 239 nm, *λ*(B(b)F and B(a)P) = 256 nm.

### 2.3. Functionalization of pectin

#### 2.3.1. Synthesis of Pec-β-CD

**Scheme 1:**
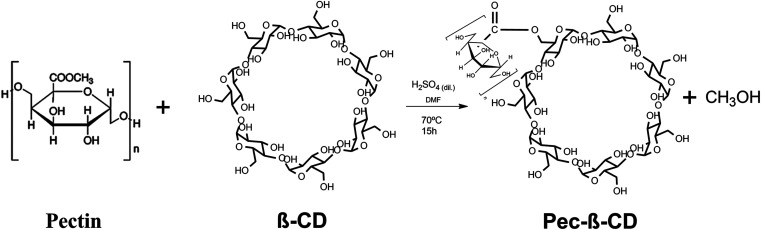
Synthetic route for the synthesis of Pec-β-CD.

Pectin modified with β-CD was prepared according to the synthetic route depicted in Scheme 1. Dried β-CD (0.1 g; 8.8 × 10^−5^ mol) and Pec (0.2 g; 3.08 × 10^−6^ mol) were dissolved in 40 mL of dry DMF and sulfuric acid (0.1 mL) was added. The reaction mixture was stirred at 70 °C for 15 hours.

After this period, the reaction mixture was cooled to room temperature and precipitated in acetone. The precipitate was separated by centrifugation, dissolved again and dialyzed in a cellulose membrane against Milli-Q© water at 25 °C. The water was renewed every 12 hours for 3 days. The Pec-β-CD (53% yield) was dried by lyophilisation for 24 hours at −55 °C on a Free Zone 4.5 Liter Benchtop vacuum freeze-drying system (USA) equipment.

#### 2.3.2. Synthesis of Pec-PVA

**Scheme 2:**
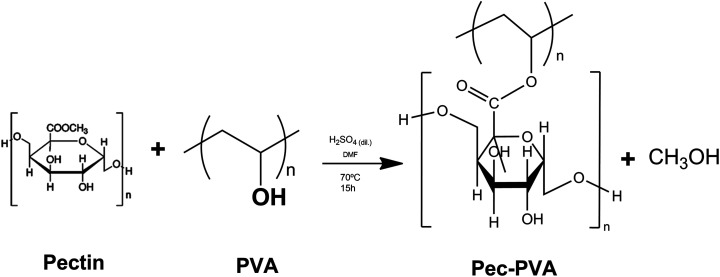
Synthetic route used for Pec-PVA synthesis.

The functionalization of Pec with PVA (see Scheme 2) was carried out by reacting PVA (0.1 g; 1.64 × 10^−6^ mol) with Pec (0.2 g; 3.08 × 10^−6^ mol) in dry DMF (40 mL), in the presence of sulfuric acid (0.1 mL). The resulting mixture was heated at 70 °C, under stirring for 15 hours.^22,32^

The reaction was cooled to room temperature; the product was precipitated by addition of excess acetone, separated by filtration, washed several times with ethanol and dialyzed in a cellulose membrane against Milli-Q© water at 25 °C. Finally, the Pec-PVA was dried by lyophilisation, for 24 hours, resulting in a 56% yield.

### 2.4. Hydrogels prepared by emulsion technique (oil-in-water)

Aqueous solution of the CS (1% w/v) was prepared in acetate buffer (1% w/w) and then filtered through a paper filter to remove insoluble substances (weight loss on the filtration process <1%). Pectin, Pec-β-CD and Pec-PVA aqueous solutions were prepared in phosphate buffer (pH 9.1) under stirring for 2 hours, at room temperature.^33^

The emulsions were obtained by mixing aqueous phase of the CS solution (1 mL) and pectin, or Pec-β-CD or Pec-PVA solution (1 mL) in a beaker with benzyl alcohol (oil phase, 5 mL) using a Ultra-Turrax at 34 × 10^3^ rpm for 5 minutes.^33^ From all tested formulations (Table 1) only those corresponding to 1 : 1 ratio of CS : Pec blends formed hydrogels.

**Table:** Formulations used for preparing different emulsions

Samples	CS (1% w/v) in acetate buffer (%)	Pec (1% w/v) in pH 9.1 buffer (%)	Pec-β-CD (1% w/v) in pH 9.1 buffer (%)	Pec-PVA (1% w/v) in pH 9.1 buffer (%)	Benzyl alcohol (v/v) (%)
Pec/CS (1 : 1)	12.5	12.5	0	0	75
Pec/CS (1 : 2)	8.3	16.7	0	0	75
Pec/CS (2 : 1)	16.7	8.3	0	0	75
Pec-β-CD/CS (1 : 1)	12.5	0	12.5	0	75
Pec-β-CD/CS (1 : 2)	8.3	0	16.7	0	75
Pec-β-CD/CS (2 : 1)	16.7	0	8.3	0	75
Pec-PVA-CS (1 : 1)	12.5	0	0	12.5	75
Pec-PVA/CS (1 : 2)	8.3	0	0	16.7	75
Pec-PVA/CS (2 : 1)	16.7	0	0	8.3	75

The resulting hydrogel blends were washed with acetone five times to remove unreacted substances and dried in desiccators at room temperature for 24 hours. The hydrogels were labelled according to their composition as Pec/CS or Pec-β-CD/CS or Pec-PVA/CS as described in Table 1.

### 2.5. Characterization of modified pectin and hydrogels

The synthesized Pec-β-CD and Pec-PVA and the hydrogels (Pec/CS, Pec-β-CD/CS and Pec-PVA/CS) have been characterized by different techniques.

Attenuated reflection infrared spectroscopy (ATR-FTIR) was performed in a Varian Cary 630 FTIR Spectrometer, with wavenumber ranging from 650 to 4000 cm^−1^.

Thermograms were obtained in a TG209 F3 Tarsus thermogravimetric analyzer (Netzsch Instruments). Samples (*ca.* 10 mg) were weighed in alumina pans and heated from 30 °C to 900 °C at a heating rate of 10 °C min^−1^ under N_2_ atmosphere (flow rate of 20 mL min^−1^).

Surface morphologies of hydrogel samples were observed by scanning electron microscopy, using a Tescan-VEGA3 SEM. For this purpose, samples were previously frozen at −20 °C and then lyophilized (Free Zone 4.5-Labconco) before being sputter-coated with a thin gold-layer.

Hydrogen nuclear magnetic resonance (^1^H NMR) spectra of Pec-β-CD, Pec-PVA and their precursors (Pec, PVA and β-CD), were recorded on Bruker Avance III 400 NMR spectrometer by dissolving the samples in deuterium oxide (D_2_O, isotope substitution > 99.9% from Eurisotop). TSP ((3-(trimethylsilyl)-2,2′,3,3′-tetradeuteriopropionic acid), EurisoTop, at concentration < 1 μM, was used as internal reference. In particular, for the PVA and Pec-PVA analysis, about 7 mg of sample were dissolved in 0.7 mL D_2_O.

The swelling (equilibrium and kinetics) of the hydrogels in water were studied by measuring the mass of swollen hydrogel and xerogel (*m*_*t*_ and *m*_*x*_, respectively), at different times, *t*, by using eqn (1)^34^ and following the procedure described in a previous work.^35^1
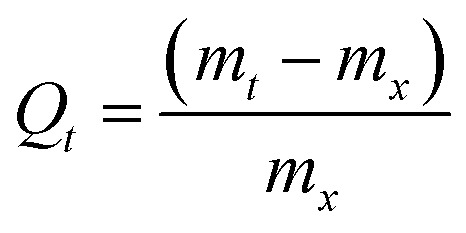


### 2.6. Sorption studies

Stock solutions containing a mixture of BTXs and PAHs were prepared by dissolving appropriated amounts of different analytes in methanol; the concentration of each analyte in that solution was 1000 mg L^−1^. The resulting solution was stored in an amber glass at −20 °C. The stock solution was then diluted with MeOH : H_2_O (70 : 30 v/v)) mixture in order to prepare the working solutions (Tables S2 and S3†).

#### 2.6.1. Sorption isotherms

The sorption isotherms of the BTXs and PAHs contaminants onto blend hydrogels were determined using batch tests. For that, 40 mL of BTXs and PAHs mixed solutions at different concentrations (Table S2†), were kept in contact during *ca.* 20 hours with the adsorbents (Pec/CS, Pec-β-CD/CS or Pec-PVA/CS) (*ca.* 4 mg) in 50 mL flasks. These mixtures were maintained at 25 °C by using a thermostatic bath (Velp Scientifica) and under continuous stirring (450 rpm). The flasks were kept closed in order to avoid the hydrocarbon volatilization and covered with aluminum sheets to avoid the oxidation and photodegradation of PAHs.^36^ To avoid the hydrogel dispersion, nylon tea-bags (100-mesh nylon screen) for avoiding the hydrogel dispersion were used.^37^ Isotherms curves were obtained in duplicate.

The concentration of hydrocarbons sorbed by the hydrogel (*q*_e_) was obtained as mass of the adsorbate removed per unit mass of the adsorbent (mg g^−1^), calculated according to eqn (2):^38^2
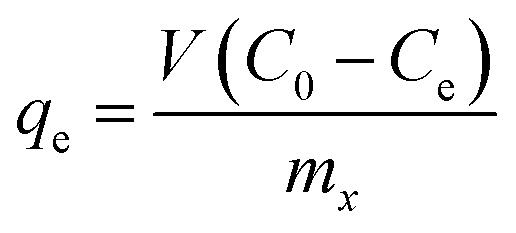
where *V* is the volume of the solution, and *C*_0_ and *C*_e_ are the initial and equilibrium concentrations (in mg L^−1^) of the analytes (BTXs and PHAs) in mixed solutions, respectively.

The removal efficiency (RE) was computed according to the following equation:^39^3
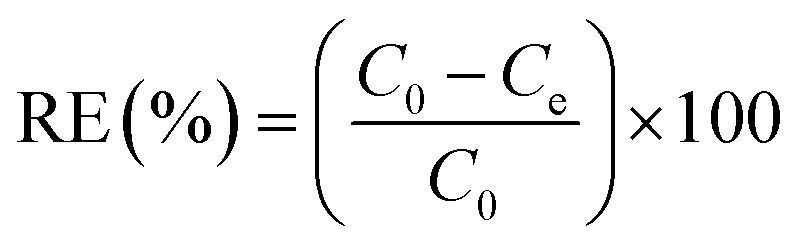


The capacity of hydrogels for the removal of BTXs and PAHs existing in a real gasoline sample was also evaluated. For that, hydrogels (*ca.* 4 mg) supported by a nylon tea-bag were introduced in a screw-caps glass tube. After that, a MeOH : H_2_O mixture (70 : 30 v/v), commercial gasoline diluted in methanol (*ca.* 1500 times) and BTXs and PAHs standard solutions at average concentration 3 mg L^−1^ and 0.7 mg L^−1^, respectively, were added, with a final volume of 40 mL. The mixture was stirred at 25 °C during one day. The amount of the BTXs and PAHs adsorbed onto blend hydrogels, in each run, was determined by measuring the concentration of those analytes occurring in the supernatant solution, before and after sorption process.

#### 2.6.2. Reusability of blend hydrogels

In order to determine the potential reusability of the hydrogels, consecutive sorption–desorption cycles were repeated two times following the procedure described above. For the desorption stage, hydrogel samples (Pec/CS, Pec-β-CD/CS or Pec-PVA/CS), previously loaded with BTX and PAHs, were collected and transferred to glass tubes with 40 mL of a MeOH : H_2_O (70 : 30 v/v) mixture, acidified to pH 3 (using HCl), and left stirring (at 450 rpm) for 5 days, at 25 °C (labeled as Des. 1). The concentrations of BTXs and PAHs were then measured by HPLC and the desorption ratio (DR) was calculated according to the following equation4
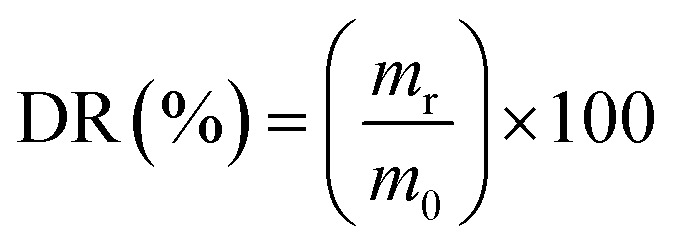
where *m*_r_ and *m*_0_ are the amounts of BTXs and PAHs desorbed and sorbed, respectively.

The blend hydrogels used in the desorption process (Des. 1) were put in contact again with the BTXs and PHAs mixed solutions in MeOH : H_2_O (70 : 30 v/v), as described above. After five days of contact, a new desorption process (Des. 2) was finalized and the concentrations of BTXs and PAHs were obtained using the same procedure as previously described for Des. 1.

#### 2.6.3. Sorption kinetics

The kinetics of sorption of BTXs and PAHs, at different concentrations (Table S3†), has been evaluated by using the following experimental procedure: the hydrogel samples were initially immersed in solutions containing BTXs and PAHs (*t* = 0); at defined intervals, *t*, aliquots of the supernatant (1 mL) were collected, filtered with PTFE in-line filter (pore size of 0.45 μm), and replaced by an equal volume of a mixture MeOH : H_2_O (70 : 30 v/v). The amount of analyte sorbed was calculated by subtracting the amount of each analyte at *t* = 0 and at time *t*, measured by HPLC in the liquid phase, and after correction of the diluting effect. The system was held at 25 °C into a thermostatic bath (Multistirrer 6 from Velp Scientifica) under constant stirring (450 rpm); other experimental details were similar to those described for sorption isotherms.

### 2.7. Post-sorption characterization of hydrogels

The effect of BTXs and some PAHs in the hydrogels (Pec/CS, Pec-β-CD/CS and Pec-PVA/CS) has been evaluated by ATR-FTIR, thermogravimetric analysis and scanning electron microscopy, using the same procedure as described in Section 2.5.

## 3. Results and discussion

### 3.1. Synthesis of Pec-β-CD and Pec-PVA

**Fig. 1:**
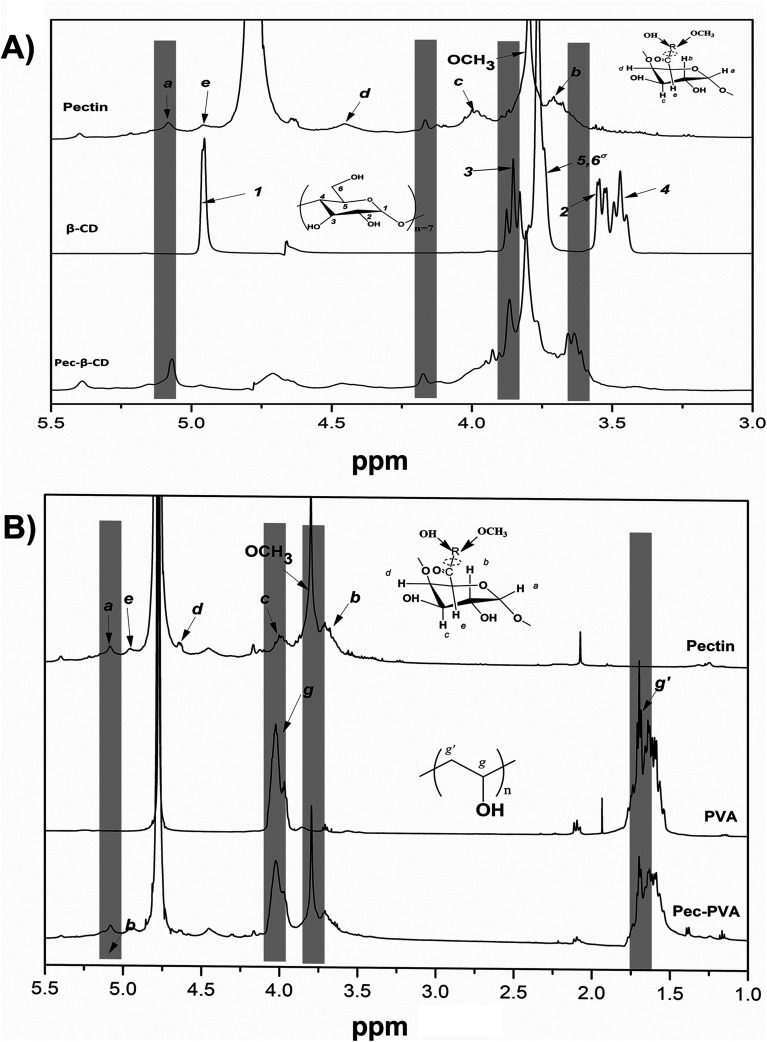
^1^H NMR spectra (D_2_O) of (A) pectin, β-CD and Pec-β-CD and (B) pectin, PVA (10% w/v in D_2_O) and Pec-PVA.

The pectin functionalization was evaluated by ^1^H NMR (Fig. 1). In the case of functionalization of Pec with β-CD (Fig. 1A), the ^1^H NMR spectrum of β-CD shows a doublet at *δ* 4.99 ppm assigned to H_1_ atoms (located outside the cavity between H_4_ and H_2_ atoms). Furthermore, the resonances occurring at *δ* 3.77–3.88 ppm are assigned to H_3_ atoms located at the wide side of the cavity.^40^ The overlapping resonances for the atoms H_5_, H_6′_ and H_6′′_ are found at *δ* 3.66–3.76 ppm.^40,41^ Resonances at *δ* 3.64, *δ* 3.86, *δ* 4.17 and *δ* 5.07 ppm that can be visualized in ^1^H NMR spectrum of Pec-β-CD (Fig. 1A) were assigned to pectin backbone. Resonances at *δ* 3.50–3.60 ppm and *δ* 3.80–3.90 ppm were also observed in such spectrum and are due to β-CD. These findings proved that the expected chemical modification effectively occurred in the structure of pectin with the insertion of β-CD.

The two broad multiplets at *δ* 1.45–1.81 ppm and *δ* 3.9–4.1 ppm present in PVA ^1^H NMR spectrum shown in Fig. 1B, were assigned to PVA polymer hydrocarbon backbone (H_g_, H_g′_–CH_2_–CH–(OH)–)_*n*_.^42^ Moreover the resonances at *δ* 1.50–1.70 ppm and *δ* 3.90–4.10 ppm in the spectra of Pec-PVA (Fig. 1B) were ascribed to PVA polymer hydrocarbon backbone. The resonances at *δ* 3.60–3.90 and 4.90–5.10 ppm in such spectrum, which correspond to pectin backbone, proved the addition of the PVA chains in the pectin structure.

In both cases, the composition of the modified pectin were also estimated from ^1^H NMR spectra, using the equation defined in a previous work.^43^ Pectin-β-CD was modified with ∼20% of β-CD and Pec-PVA has ∼17% of pectin.

**Fig. 2:**
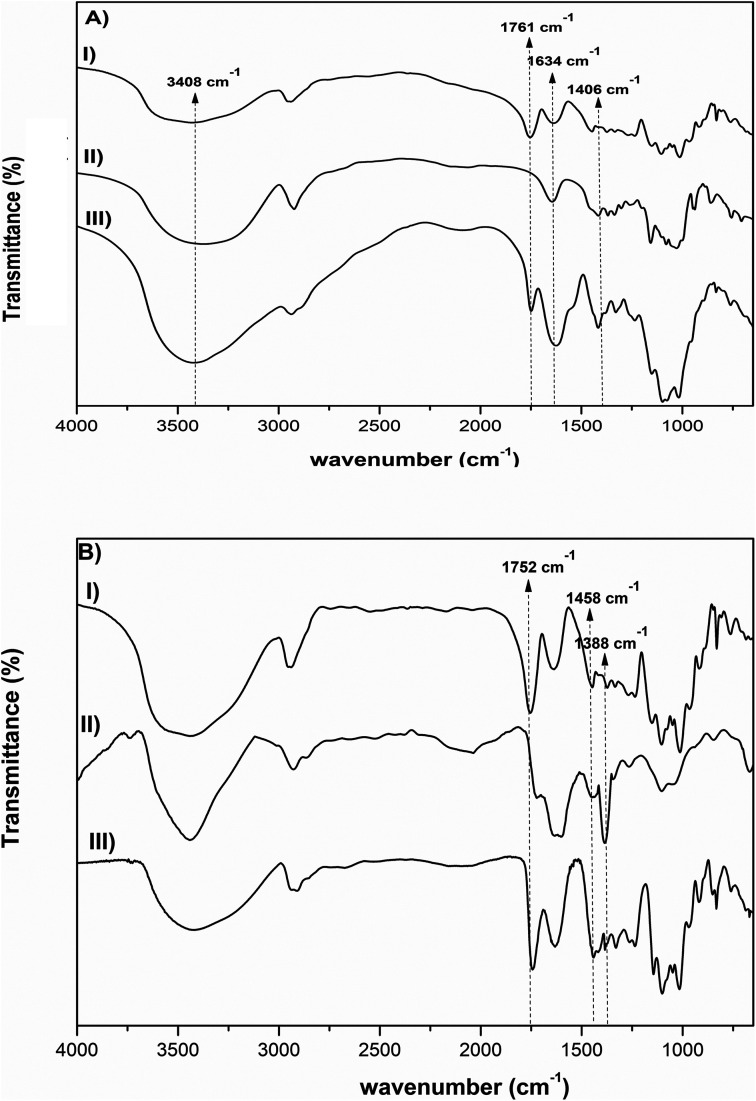
FTIR spectra of (A) pectin, β-CD, Pec-β-CD (I, II and III, respectively), and (B) pectin, PVA, Pec- PVA (I, II and III, respectively).

FTIR analysis was also done in order to further investigate the interactions between the polysaccharides. Fig. 2A shows the FTIR spectra of pectin, β-CD and Pec-β-CD. These spectra are characterized by the following vibrational bands: (i) the strong band at 3408 cm^−1^ was assigned to –OH stretching vibration; (ii) the peak at 2943 cm^−1^ was attributed to the methylene stretching vibrations of the alkyl chains of the polysaccharides; (iii) and the most significant bands relative to the pectin can be observed at 1761 cm^−1^, originated by C=O stretching, at 1634 cm^−1^ (due the asymmetric stretching of C=O), and at *ca.* 1000 cm^−1^ (the finger-print region, 1200–800 cm^−1^), typical of pectin polymers and assigned to the C–O stretching.^44,45^ For Pec-β-CD, a characteristic peak at 1761 cm^−1^ assigned to the C=O of pectin as well as a peak at 1406 cm^−1^ due to β-CD can be clearly observed. The intensity of the peaks at 3408, 1634 and 1406 cm^−1^ increased upon β-CD incorporation, indicating the formation of new covalent bonds.^46^

The FTIR spectrum of PVA (Fig. 2B) shows several characteristic bands: a broad band at 3800–3200 cm^−1^ assigned to the –OH vibration, the C–H stretching vibration at 2926 cm^−1^, the sharp band at 1635 cm^−1^ corresponds to the C–O stretching, and the band observed at 1355 cm^−1^ has been attributed to combination frequencies of CH–OH. These vibrational modes are also present in pectin and Pec-PVA FTIR spectra.

The FTIR spectrum of Pec-PVA showed a peak at 1752 cm^−1^, which was related to the stretching of the C=O bonds, that is not present in the PVA spectrum. It is also possible to note a higher intensity in the peaks at 1458 and 1388 cm^−1^ as compared to the pectin FTIR spectrum, thus evidencing the presence of both polymers in Pec-PVA, as expected.^47,48^ Both FTIR spectroscopy and ^1^H NMR revealed pectin modification.

### 3.2. Hydrogels characterization

**Fig. 3:**
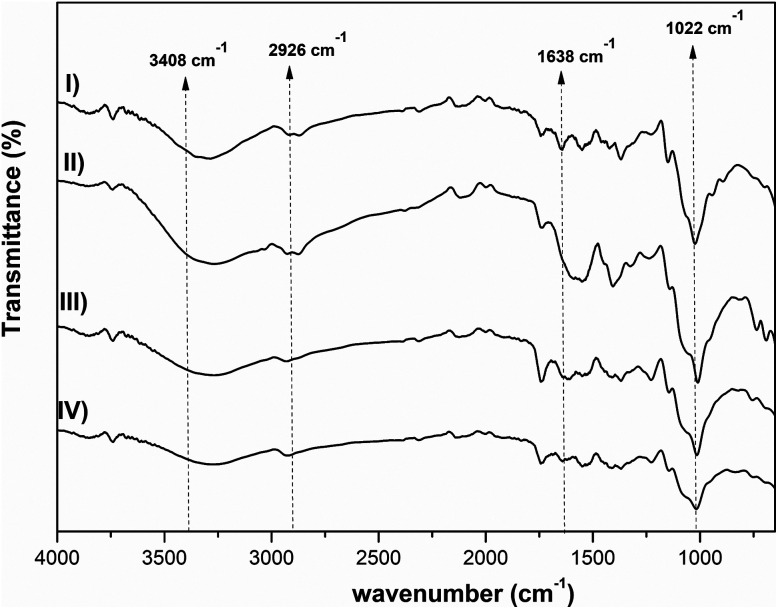
FTIR spectra of CS, Pec/CS, Pec-β-CD/CS, Pec-PVA/CS hydrogels (I, II, III and IV), respectively.

Fig. 3 shows the ATR-FTIR spectra of the three different synthesized hydrogels. The spectra are characterized by the following vibrational bands: the strong band at 3408 cm^−1^ is assigned to the hydroxyl stretching vibration of the polysaccharides; the broad band/shoulder at 3355–3300 cm^−1^ can be assigned to the –OH and –NH stretching of PVA and chitosan; the C–H stretching vibration is observed by the band at 2926 cm^−1^; the vibrational mode at 1638 cm^−1^ is due to –C=O stretching; the band at 1420 cm^−1^ was ascribed to C–H deformation vibration; and the bands at *ca.* 1022 cm^−1^, in the finger-print region (1200–800 cm^−1^), are typical of pectin polymers and can be assigned to the C–O stretching.^44^

**Fig. 4:**
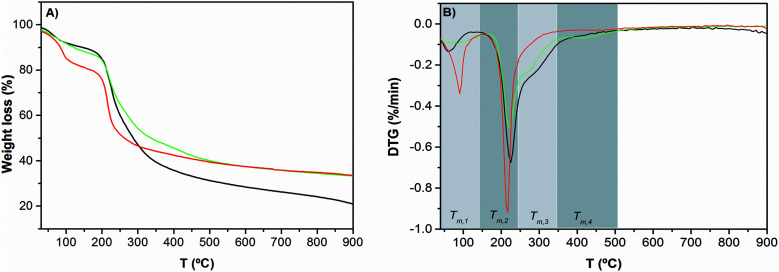
TGA curves (A) and DTG curves (B) of Pec/CS (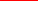), Pec-β-CD/CS (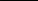) and Pec-PVA/CS (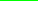). See the text for the meaning of *T*_m,*i*_.

The effect of pectin functionalization on the thermal stability of hydrogels was evaluated by thermogravimetric analysis (Fig. 4).

*T*_m,*i*_ is the maximum degradation rate at temperature range *i*. By comparing the *T*_m,*i*_ for functionalized and non-functionalized blends, for a giving temperature range *i*, it can be concluded that the incorporation of PVA or β-CD in pectin has a significant effect on the thermal behavior of modified pectin/CS blends. For the first degradation step (*T*_1_), the functionalization of pectin leads to a smaller water weight loss, from 19% (for Pec/CS) to 12% (for Pec-β-CD/CS) and 10% (for Pec-PVA/CS) and a lower *T*_m,1_, from 90 to 62 °C and 83 °C, respectively. Concerning the main degradation step (*T*_m,2_), it has been found that for Pec/CS: *T*_m,2_ = 217 °C. This maximum degradation temperature was essentially assigned to pectin degradation (235 °C).^49^ The difference between such temperatures was due to the formation of the blend by coacervation. It is also worth noticing that the thermal degradation step assigned to chitosan (*T* = 310 °C)^50^ was detected for this blend, as shown in DTG curves (Fig. 4B). However, the previous functionalization of pectin before the formation of the blend gels leads to a decrease in electrostatic interactions between the positively charged chitosan and the negatively charged pectin, inducing some phase separation. Consequently, the *T*_m,2_ for both blends (225 and 224 °C for β-CD- and PVA-modified pectin-containing blends, respectively) approaches the value for pectin. This is accompanied by the occurrence of shoulders in the DTG curves at temperatures around 276 °C (*T*_m,3_), which might be related with the chitosan degradation temperature; *i.e.*, the functionalization of pectin makes the blend more heterogeneous. Further degradation step for the PVA-containing gel was found at *T*_m,4_ = 419 °C and was ascribed to PVA and suggests a loss of the PVA crystallinity.^27^

**Fig. 5:**
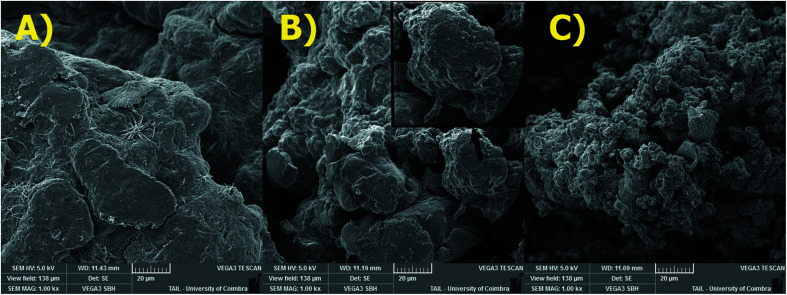
SEM images of: (A) Pec/CS; (B) Pec-β-CD/CS and (C) Pec-PVA/CS hydrogels (magnification: ×1000). Scale = 20 μm.

The surface morphology of adsorbents was also studied by SEM (Fig. 5). The SEM image for PEC/CS blend shows a featureless morphology. However, significant differences in the surface morphology occur if β-CD- and PVA-functionalized pectin was used to prepare the blend. The surface β-CD-containing blend is more rough and heterogeneous and, when the cross section is visible, there are indications that lamellar structure (see zoom in insight picture – Fig. 5B) was formed; on the other hand, the Pec-PVA/CS surface is more irregular and exhibits granule-like structure.

### 3.3. Swelling degree

**Fig. 6:**
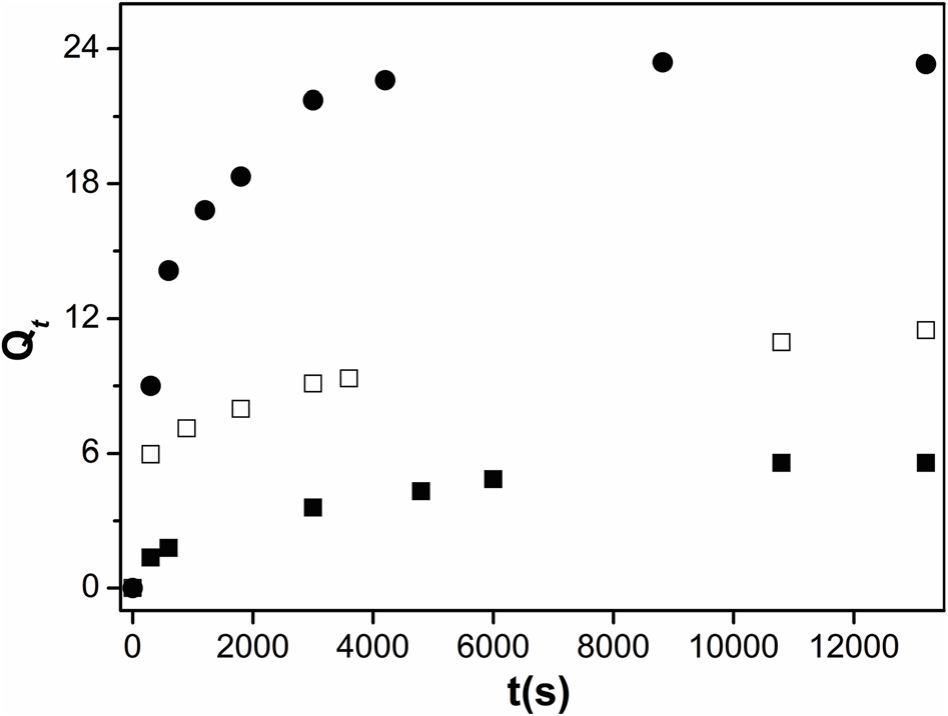
Swelling kinetics of Pec/CS (■), Pec-β-CD/CS (□) and Pec-PVA/CS (●) hydrogels in water, at 25 °C.

The effect of pectin functionalization on the structure of blend hydrogels was further analyzed by measuring the swelling degree at 25 °C (Fig. 6). The presence of CD or PVA on modified-pectin strongly affects the gels' ability to swell (see *Q*_e_ values in Table 2) in agreement with data obtained from TGA and SEM images. Although both CD and PVA possess characteristic amphiphilic features,^27,51^ it should be expected that PVA would contribute to the occurrence of a more heterogeneous and porous blend and, consequently, its hydrophilicity was enhanced because it showed higher swelling capacity (4-fold higher than for Pec/CS).

**Table:** Kinetic parameters for the swelling of water by the Pec-/CS based blends gels, at 25 °C

	Eqn (5)			Eqn (6)		
	*Q*_e,exp_	*k*_1,w_ (10^−5^ s^−1^)	AIC	*Q*_e_	*k*_2,w_ (10^−4^ s^−1^)	AIC
Pec/CS	5.6 (±0.3)	1.3 (±0.1)	3.60	5.6 (±0.2)	1.5 (±0.9)	9.52
Pec-β-CD/CS	11.5 (±0.6)	1.2 (±0.1)	2.94	11.9 (±0.3)	1.2 (±0.3)	7.90
Pec-PVA/CS	23 (±1)	2.04 (±0.02)	4.15	23 (±1)	0.68 (± 0.02)	6.35

In order to get an insight on the water sorption mechanism, the swelling kinetics was evaluated using first- and second-order kinetic equations,^52^ by using the following linearized equations, respectively:5
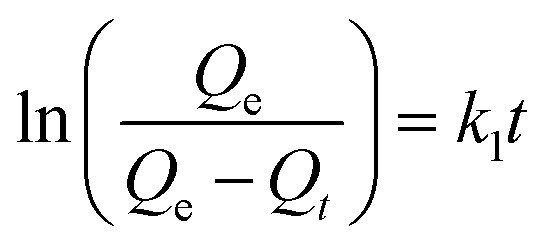
6
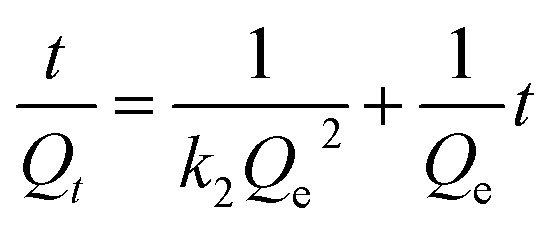
where *k*_1_ and *k*_2_ are the swelling rates, and *Q*_*t*_ and *Q*_e_ are the swelling ratios at time *t* and equilibrium conditions. The best model has been chosen through the Akaike's information criteria (AIC) by using the following equation:^53^7
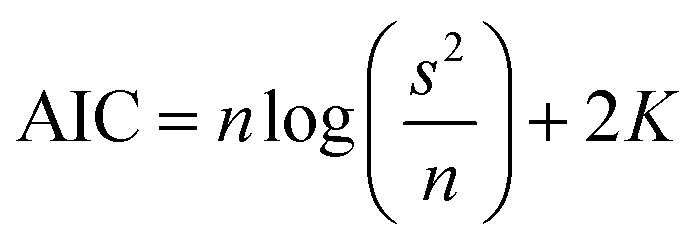
where *s*^2^ is the residual sum of squares, *n* is the number of experimental data points and *K* is the number of model parameters. The fitting of eqn (5) and (6) to the experimental data (*Q*_*t*_/*Q*_e_ < 0.9) (see Table 2) shows that, for all blends, the swelling kinetics follows a first order kinetic mechanism. This shows that water–water interactions are stronger than water–polymer interactions; however, for the most swollen hydrogel, the rate constant is considerably higher. Taking into account that the sorption of water is driven by dipole–dipole and hydrogen-bonding interactions, it can be hypothesized that, in this particular case, interactions between hydroxyl groups of PVA and water molecules are stronger than those occurring with other polymers.^52^

### 3.4. Sorption kinetics

**Fig. 7:**
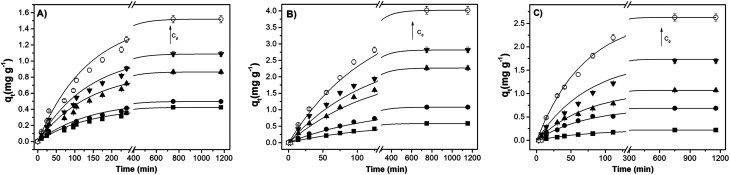
Sorption kinetic of benzene, in BTXs and PAHs mixed solutions, onto (A) Pec/CS, (B) Pec-β-CD/CS and (C) Pec-PVA/CS hydrogels, at 25 °C and for several benzene *C*_0_ values. The arrow indicates the direction of higher concentrations (for further details see Table S3†). The solid lines are just a guide for the eyes.

The sorption kinetics allows an evaluation concerning the time necessary to attain the sorption equilibrium as well as to give an insight on the kinetic mechanism. Fig. 7 shows representative sorption kinetics of benzene present, in BTXs and PAHs mixed solutions, onto Pec/CS, Pec-β-CD/CS and Pec-PVA/CS hydrogels, at 25 °C. On average, the time needed for reaching the equilibrium ranged from 2.6 to 4.1 hours, depending on the initial concentration *C*_0_ of adsorbate.

For such multi-compound systems, the adsorption can be affected by different factors, such as adsorbent–adsorbate and adsorbate–adsorbate interactions, and gel swelling. In these circumstances the modelling of sorption kinetics is better suited by the pseudo-first-order and pseudo-second-order kinetic models,^54,55^ since they only take into account the effect of the overall measured macroscopic parameter on the integral sorption rate. These models can be described, respectively, by eqn (5) and (6) where *Q*_e_ and *Q*_*t*_ must be replaced by *q*_*t*_ and *q*_e_ (both in mg g^−1^) – the amounts of the adsorbate sorbed at time *t* and at equilibrium, respectively.

The fitting parameters of eqn (5) and (6) to experimental sorption data for BTXs and PAHs, and the corresponding AIC, are reported in the ESI (Fig. S1–S3 and Tables S4–S6†). The sorption kinetics for all adsorbates follows a first-order kinetic model. This model suggests that the sorption is a diffusion-controlled process, in line with the fact that physisorption is the rate limiting phenomenon in the sorption mechanism, being characterized by the occurrence of a multilayer sorption and by semi-reversible sorption/desorption cycles.^18,56^ This will be discussed in the following sections.

### 3.5. Sorption isotherms

Fig. S4† shows the sorption isotherms for all six aromatic compounds sorbed by the three blend hydrogels. From a general overview, it can be concluded that the sorbed amount of adsorbate increases by increasing the initial concentration, suggesting that the mass transfer is driven by the concentration gradient; these results seem to be in agreement with those reported by Mohamed and Ouki^18^ for the sorption of toluene (with concentration ranging from 5 to 100 mg L^−1^) by chitosan (15 g L^−1^). It can also be noticed that in all cases the sorption process is cooperative, which agrees with the occurrence of multilayer adsorption characterized by short-range interactions, in agreement with the discussion carried out in the previous section.

In order to go deeper on the sorption mechanism, Freundlich^57^ and BET^58^ models have been fitted to the experimental sorption data. These models can be expressed by using the following equations, respectively,

8 *q*_e_ = *K*_F_*C*_e_^1/*n*_F_^

9
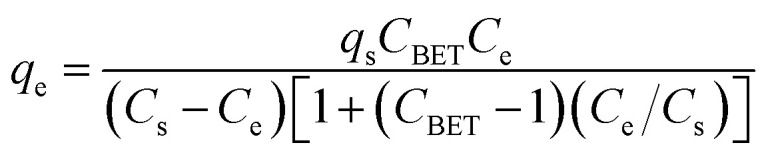


In eqn (8), *K*_F_ is the Freundlich isotherm constant, indicative of the relative sorption capacity and *n*_F_ is a constant related with surface heterogeneity.^59^ In eqn (9), *C*_BET_, *C*_s_ and *q*_s_ are the BET adsorption isotherm parameters, related to the energy of surface interaction, adsorbate monolayer saturation concentration, and theoretical isotherm saturation capacity, respectively.^57^Table 3 summarizes the fitting parameters computed from the non-linear fitting^58^ of eqn (8) and (9) to experimental data (Fig. S4†), by using OriginLab software.

Freundlich and BET parameters obtained by fitting eqn (8) and (9) to the experimental data (Fig. S4)^a^

**Table:** 

		Freundlich			BET			
Pec/CS	*C*_0_ (mg L^−1^)	*K*_F_ (mg^(*n*−1)/*n*^ L^1/*n*^ g^−1^	1/*n*_F_	*R* ^2^	*q*_s_ (mg g^−1^)	*C*_BET_	*C*_s_ (mg L^−1^)	*R* ^2^
Benzene	11.5–53.1	0.02 (±0.01)	1.1 (±0.2)	0.9055	0.42 (±0.04)	19 (±14)	66 (±2)	0.9894
Toluene	10.5–57	0.01 (±0.01)	1.3 (±0.1)	0.9715	1.3 (±0.5)	3 (±2)	84 (±13)	0.9909
Xylenes	9.5–60.5	0.002 (±0.002)	1.8 (±0.2)	0.9565	0.99 (±0.16)	4 (±2)	73 (±3)	0.9948
Pyrene	2.1–12.5	0.03 (±0.01)	1.3 (±0.1)	0.9811		n/c		
B(b)F	2–11.5	0.01 (±0.01)	1.7 (±0.2)	0.9819		n/c		
B(a)P	2–13.1	0.02 (±0.01)	1.5 (±0.1)	0.9931		n/c		

a *values inside brackets are standard deviations of the average; n/c: the fit does not converge.

**Table:** 

Pec-β-CD/CS		*K*_F_ (mg^(*n*−1)/*n*^ L^1/*n*^ g^−1^	1/*n*_F_	*R* ^2^	*q*_s_ (mg g^−1^)	*C*_BET_	*C*_s_ (mg L^−1^)	*R* ^2^
Benzene	11.4–38.7	0.003 (±0.001)	2.2 (±0.2)	0.9733	1.9 (±0.6)	1.4 (±0.6)	42 (±2)	0.9974
Toluene	9.6–38.9	0.002 (±0.002)	2.3 (±0.5)	0.9069	0.7 (±0.1)	8 (±5)	38.1 (±0.7)	0.9958
Xylenes	10.5–41	0.002 (±0.001)	2.2 (±0.3)	0.9594	1.0 (±0.2)	3 (±1)	44 (±2)	0.9946
Pyrene	2.1–10.1	0.001 (±0.001)	3.3 (±0.7)	0.9321	0.14 (±0.01)	19 (±16)	9.1 (±0.1)	0.9982
B(b)F	1.5–13.5	0.002 (±0.001)	2.7 (±0.6)	0.9349	0.22 (±0.01)	5 (±1)	13.2 (±0.2)	0.9983
B(a)P	2.7–11.5	0.001 (±0.001)	2.2 (±0.4)	0.9370	0.18 (±0.01)	5 (±1)	1.6 (±0.2)	0.9989

**Table:** 

Pec-PVA/CS		*K*_F_ (mg^(*n*−1)/*n*^ L^1/*n*^ g^−1^	1/*n*_F_	*R* ^2^	
Benzene	8.8–38.6	0.01 (±0.01)	1.6 (±0.1)	0.9830	n/c
Toluene	7.7–38.1	0.01 (±0.01)	1.7 (±0.2)	0.9817	n/c
Xylenes	7.0–37.1	0.01 (±0.01)	1.7 (±0.1)	0.9899	n/c
Pyrene	1.6–7.2	0.01 (±0.02)	2.9 (±0.2)	0.9952	n/c
B(b)F	0.8–6.6	0.02 (±0.01)	2.2 (±0.1)	0.9926	n/c
B(a)P	1.3–6.1	0.01 (±0.01)	2.9 (±0.3)	0.9820	n/c

The analysis of Table 3 shows that the simultaneous sorption of BTX and PAHs significantly depends on the adsorbent. In the case of Pec-PVA/CS, the sorption of all compounds is well justified by the Freundlich equation, with a heterogeneity factor higher than 1, indicating an occurrence of multilayer physically-based sorption. This mechanism is in agreement with the ability of these sorbents to interact *via* π–π stacking interactions. However, in the case of Pec/CS blend hydrogels the mechanism is essentially different for BTXs. This clearly suggests the occurrence of a monolayer which can be justified by the interaction between these compounds and pectin and/or chitosan.^60^ Although further work should be done to unveil such mechanism, we can hypothesise that short-range (weak) interactions are behind that mechanism; in fact, the water volume fraction of PVA-containing gel is 4 times higher than the corresponding blend without PVA (*i.e.*, 0.04 and 0.16, respectively).^61^ Even so, the removal efficiency of BTXs and PAHs by PVA-containing blends is significantly higher than the RE obtained by using the Pec/CS blend (see discussion below), suggesting that the gel-phase obtained with the former gel stabilises the dissolution of all adsorbates.

For the Pec-β-CD/CS, the BET mechanism shows the better determination coefficient for all adsorbates. From the analysis of fitting parameters, it is clear that *C*_s_ values for BTXs are significantly higher than those obtained for PAHs; based on this, we can conclude that the concentration gradient and the size of adsorbates are key points on the sorption process.

**Fig. 8:**
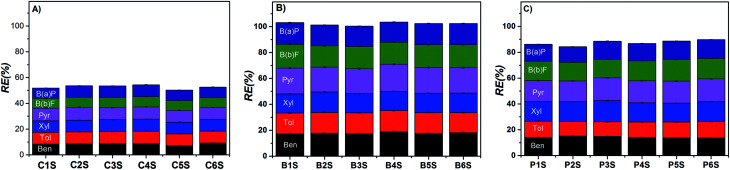
Effect of aromatic compounds concentration on the simultaneous sorption efficiency of the (A) Pec/CS, (B) Pec-β-CD/CS and (C) Pec-PVA/CS hydrogels, at 25 °C.

Furthermore, it is also interesting to find out that *C*_s_ values for BTX in the Pec/CS are, in average, 45% smaller than those computed for Pec-β-CD/CS. Since the contribution of PAHs for the total *C*_s_ is not enough to reach that value, it can be anticipated that the presence of CD is limiting the occurrence of the monolayer. However, from the analysis of the removal efficiency we can conclude that β-CD is playing a major role in the sorption process (see Fig. 8). In fact, Pec-β-CD/CS shows, by far, the highest RE (*ca.* 102%). This RE is slightly higher than that found for the simultaneous sorption of the same adsorbates onto methyl-modified silica aerogel, prepared from methyltrimethoxysilane (MTMS) precursor^62^ and two times higher than that found for Pec/CS. The performance of the Pec-β-CD/CS towards benzene (used as a model molecule) sorption was also measured for the sake of comparison with other existing adsorbents. The RE for benzene has been measured and a value of 66.1 (±0.3)% was obtained. However, this value is lower than REs reported for carbon-based materials: for example, the sorption of benzene onto granular-activated carbon^63^ or multi-walled carbon nanotubes^64^ leads to RE of approximately 90 and 97.7%, respectively.

### 3.6. Sorption–desorption cycles

The production cost and reusability of the adsorbents are the most effective parameters for the sorbents in the contaminated environment treatment systems.^65^ Furthermore sorption–desorption studies may also give complementary information on the adsorbent–adsorbate interaction mechanism.

**Fig. 9:**
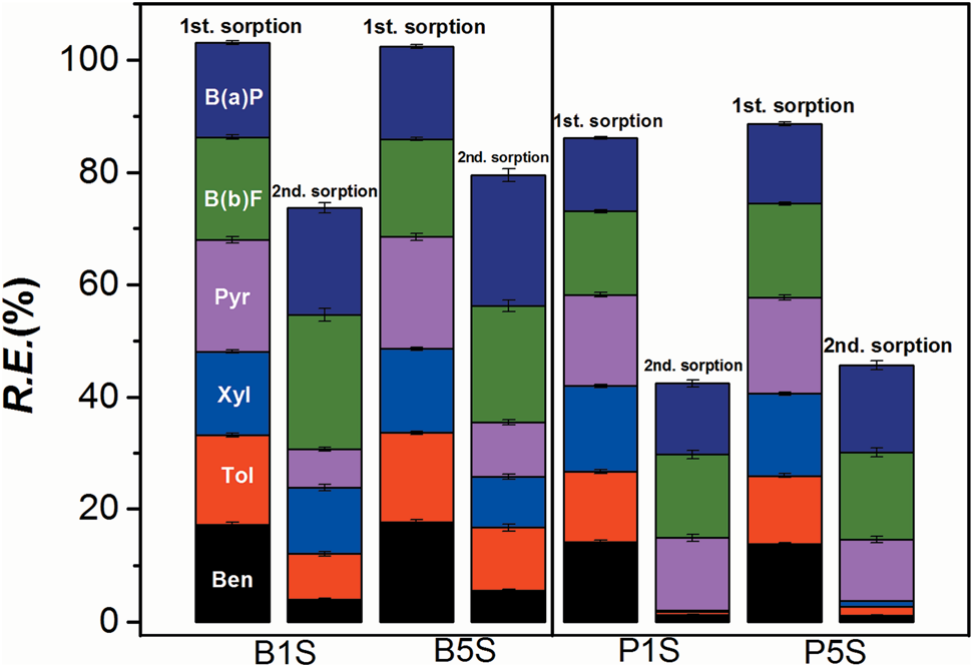
Comparative results on the removal efficiency (RE) of the BTXs and PHAs by the hydrogels (Pec-β-CD/CS (B_1_ and B_5_) and Pec-PVA/CS (P_1_ and P_5_)), after the first desorption (Des. 1). The data for the 1st sorption stage are duplicated (from Fig. 8) for the sake of comparison.

**Fig. 10:**
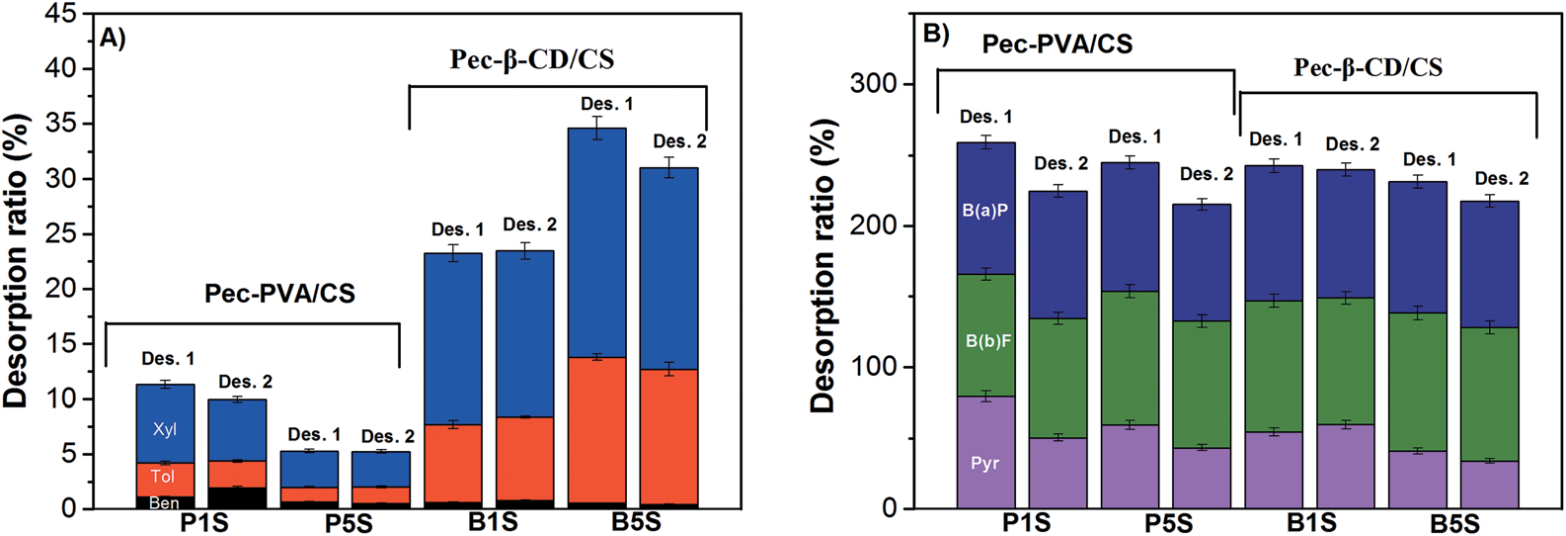
Desorption results for six miscible organics (BTXs (A) and PAHs (B)) on Pec-PVA/CS and Pec-β-CD/CS hydrogels. Temperature and contact time with the solution MeOH : H_2_O (70 : 30 v/v), respectively: 25 °C and 300 h.

Fig. 9 shows that the 2nd sorption process is relatively effective for Pec-β-CD and Pec-PVA-containing blends gels. It can also be observed that the removal efficiency for B(a)P and B(b)F does not vary from the 1st to 2nd sorption. On the other hand and in particular for β-CD-containing gel, the removal efficiency of BTXs significantly decreased. A rational for such evidences can be find from the analysis of desorption ratio (Fig. 10). B(b)F and B(a)P are significantly desorbed from hydrogels, with DR higher than 82%, whilst pyrene is only moderately desorbed with DR around 34%. On the other hand, BTXs are significantly retained by the hydrogels. Among them, benzene is the highest retained adsorbate, independently of the gel. This selectivity of both gels (Pec-PVA/CS and Pec-β-CD/CS) towards benzene is not easily justified. In fact, the association constant for β-CD-benzene is of same order of magnitude than those found for xylene;^25^ on the other side, it seems that the gel phase produced by the incorporation of PVA seems to stabilize BTXs. The latter can be understood (among other possible hypothesis) if the presence of PVA has a water-structure making^66^ in agreement with its amphiphilic properties recently discussed,^27,51^ and with the *k*_2,w_ dependence on *Q*_e_, allowing a better dissolution of benzene, and in a less degree toluene and xylene.

### 3.7. Performance of hydrogels towards water contaminated with real petroleum sample

Here we intended to demonstrate the capacity of the Pec/CS, Pec-β-CD/CS and Pec-PVA/CS blend hydrogels for efficiently remove BTXs and PAHs from a commercial gasoline sample. Table 4 summarizes the initial concentrations (*C*_0_) of BTXs and some PAHs in the sample.

**Table:** Parameters on adsorption of BTXs and PAHs in a real petroleum sample (diluted gasoline in methanol spiked with known individual amounts of BTXs and PAHs (about 3 mg L^−1^ and 0.7 mg L^−1^, respectively), and using 4 mg of hydrogel sample (Pec/CS, Pec-β-CD/CS and Pec-PVA/CS)^a^

	*C*_0_ in the used commercial gasoline-before dilution (wt%)	*C*_0_ in the spiked solution (mg L^−1^)	*q*_e,exp_ (mg g^−1^)	Removal efficiency (%)
**Pec/CS**				
Benzene	0.09 (±0.01)*	4.1 (±0.1)	0.046 (±0.001)	1.2 (±0.1)
Toluene	0.75 (±0.01)	7.4 (±0.2)	0.057 (±0.001)	0.83 (±0.04)
Xylenes	1.31 (±0.04)	11.2 (±0.4)	0.090 (±0.002)	0.87 (±0.04)
Pyrene	0.011 (±0.001)	1.3 (±0.1)	0.052 (±0.001)	4.3 (±0.3)
B(b)F	4.0 (±0.1) × 10^−4^	0.31 (±0.03)	0.007 (±0.001)	2.3 (±0.1)
B(a)P	5.0 (±0.2) × 10^−3^	0.49 (±0.02)	0.034 (±0.003)	7.5 (±0.4)

**Pec-β-CD/CS**				
Benzene	0.09 (±0.01)*	4.9 (±0.1)	0.175 (±0.003)	5.5 (±0.3)
Toluene	0.75 (±0.01)	9.4 (±0.3)	0.238 (±0.004)	3.9 (±0.2)
Xylenes	1.31 (±0.04)	15.2 (±0.5)	0.158 (±0.003)	1.6 (±0.1)
Pyrene	0.011 (±0.001)	0.97 (±0.03)	0.059 (±0.001)	9.3 (±0.5)
B(b)F	4.0 (±0.1) × 10^−4^	0.61 (±0.02)	0.036 (±0.001)	9.0 (±0.3)
B(a)P	5.0 (±0.2) × 10^−3^	0.99 (±0.03)	0.066 (±0.001)	10.2 (±0.5)

**Pec-PVA/CS**				
Benzene	0.09 (±0.01)*	3.9 (±0.1)	0.119 (±0.003)	3.4 (±0.2)
Toluene	0.75 (±0.01)	8.3 (±0.2)	0.164 (±0.003)	2.2 (±0.2)
Xylenes	1.31 (±0.04)	13.0 (±0.4)	0.129 (±0.004)	1.1 (±0.1)
Pyrene	0.011 (±0.001)	0.49 (±0.01)	0.026 (±0.001)	5.8 (±0.3)
B(b)F	4.0 (±0.1) × 10^−4^	0.11 (±0.01)	0.007 (±0.001)	6.9 (±0.2)
B(a)P	5.0 (±0.2) × 10^−3^	0.33 (±0.01)	0.025 (±0.001)	8.5 (±0.3)

a *values inside brackets are standard deviations of the average.

The analysis of simultaneous sorption demonstrates that the cumulative removal efficiencies of BTXs and PAHs are, respectively 2.9 and 14.1% by Pec/CS hydrogel; 11 and 28.5% by Pec-β-CD/CS hydrogel; 6.7 and 21.2% by Pec-PVA/CS hydrogel. Thus, we noticed that in the real samples the removal efficiency for BTXs is much lower than the corresponding value for PAHs in all used hydrogels. This low BTXs removal efficiency may have been influenced by the diverse number of monoaromatic compounds present in commercial gasoline.^67^

As shown, the presence of β-CD and PVA in the hydrogel significantly increased the removal efficiency of the hydrocarbons by the studied materials. This behaviour is in agreement with data showed in the previous sections.

## 4. Conclusions

The β-CD- and PVA-Pec/CS composite gels show a 2-fold and 4-fold higher swelling degree, respectively, than the unmodified Pec/CS hydrogel. In both cases the grafting of those compounds originates a decrease in the Pec/CS electrostatic interactions, leading to an increase in the plasticity of the polymeric structure and a decrease in the thermal degradation temperature. The hydrogel with intermediate swelling features shows the best removal efficiency for BTXs and PAHs. Whilst PVA has a major role on the plasticizing of polymer structure, the incorporation of β-cyclodextrin shows a higher efficiency for the sorption process. The latter is probably due to the ability of CD to form host-guest supramolecular structures with aromatic moities. The cumulative RE of gels towards polycyclic aromatic compounds are 53 (±4)%, 102 (±10)% and 87 (±10)%, to Pec/CS, Pec-β-CD/CS and Pec-PVA/CS hydrogels, respectively. The RE is not dependent on the initial concentration of adsorbates. The sorption process occurs through a multilayer, non-selective mechanism, in the three different gels. The sorption kinetics follows a 1st order kinetics, suggesting that the formation of the monolayer is faster than the subsequent multilayer. The PAHs are more easily desorbed than the BTXs. This can be justified by an initially sorption of BTXs at the hydrogel interface. The performance of these Pec/CS based gels for the removal of BTXs and PAHs from a real sample of gasoline has been tested. Despite the high complexity of the mixture, the REs of the six different adsorbates show non-negligible values for both composite blends; this provides good clues for the development of biogels to address environmental issues.

## Conflicts of interest

There are no conflicts to declare.

## Supplementary Material

RA-008-C8RA02332H-s001
