# Supplementary material for: Synthesis, characterization and sorption studies of aromatic compounds by hydrogels of chitosan blended with β-cyclodextrin- and PVA-functionalized pectin
Source: RSC Adv. 2018 Apr 18;8(26):14609–22. doi: 10.1039/c8ra02332h (PMC9079937; doi:10.1039/c8ra02332h)
Supplement: RA-008-C8RA02332H-s001 [file RA-008-C8RA02332H-s001.pdf]

Electronic Supplementary Material (ESI) for RSC Advances.
This journal is © The Royal Society of Chemistry 2018

Electronic Supplementary Information

Synthesis, characterization and sorption studies of aromatic compounds by hydrogels of chitosan blended with β-cyclodextrin- and PVA-functionalized pectin

Cesar M. C. Filho[1], Pedro V. A. Bueno[2], Alan F. Y. Matsushita[1], Adley F. Rubira[2] Edvani C. Muniz[2,3], Luísa Durães[4], Dina M. B. Murtinho[1] and Artur J. M. Valente[1,*]

[1]*CQC, Department of Chemistry, University of Coimbra, 3004-535 Coimbra, Portugal.*

[2]*Grupo de Materiais Poliméricos e Compósitos (GMPC) - Departamento de Química, Universidade Estadual de Maringá, UEM, 87020-900, Maringá, PR, Brazil.*

[3]*Post-graduate Program on Materials Science & Engineering, Federal University of Technology, Paraná (UTFPR-LD), 86036-370, Londrina, PR, Brazil.*

[4]*CIEPQPF, Department of Chemical Engineering, University of Coimbra, Rua Sílvio Lima 3030-790 Coimbra, Portugal*

**Corresponding author*

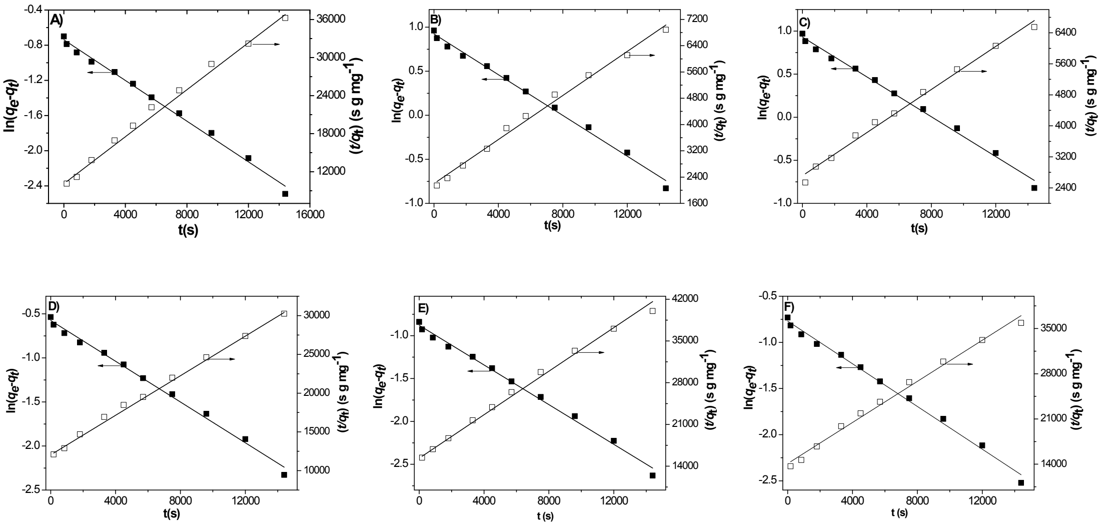

Figure S1. Representative plots of the fitting of linearized forms of pseudo-first (■) and pseudo-second (□) order equations to experimental sorption data for (A) Ben (B) Tol (C)Xyl (D) Pyr (E) (B(b)F and (F) B(a)P onto the Pec/CS hydrogel, at 25 ºC.

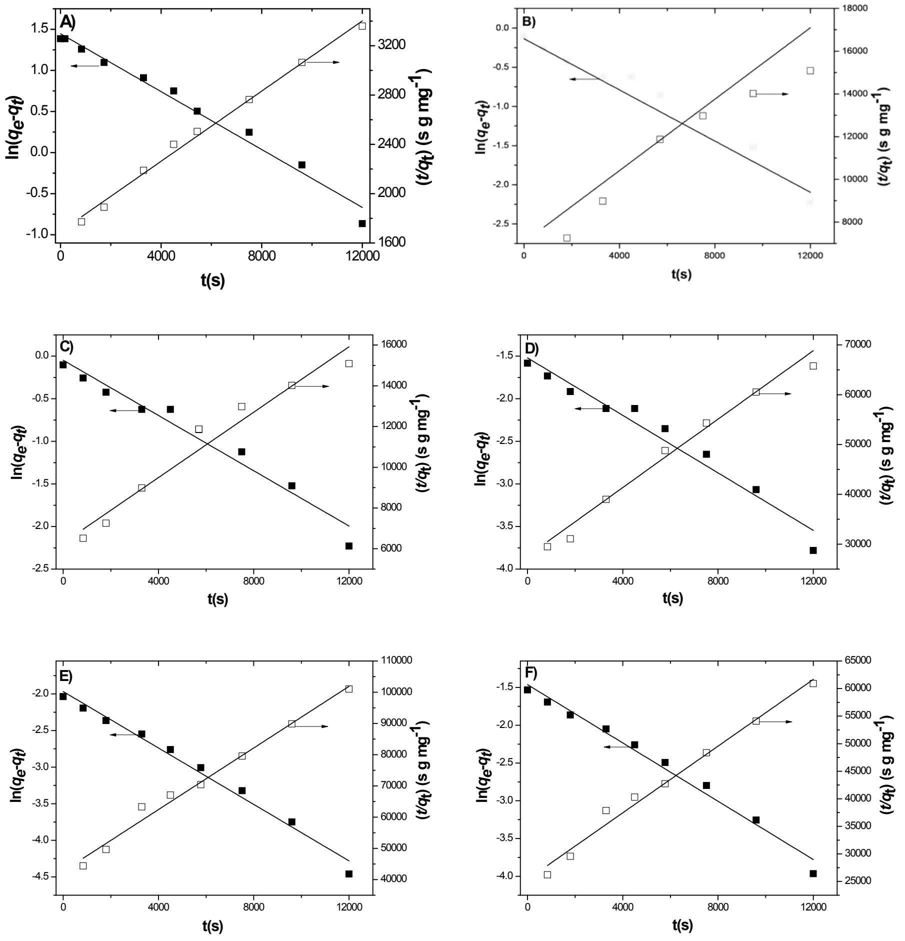

Figure S2. Representative plots of the fitting of linearized forms of pseudo-first (■) and pseudo-second (□) order equations to experimental sorption data for A) Ben (B) Tol (C) Xyl(D) Pyr (E) (B(b)F and (F) B(a)P onto the Pec-β-CD/CS hydrogel, at 25ºC.

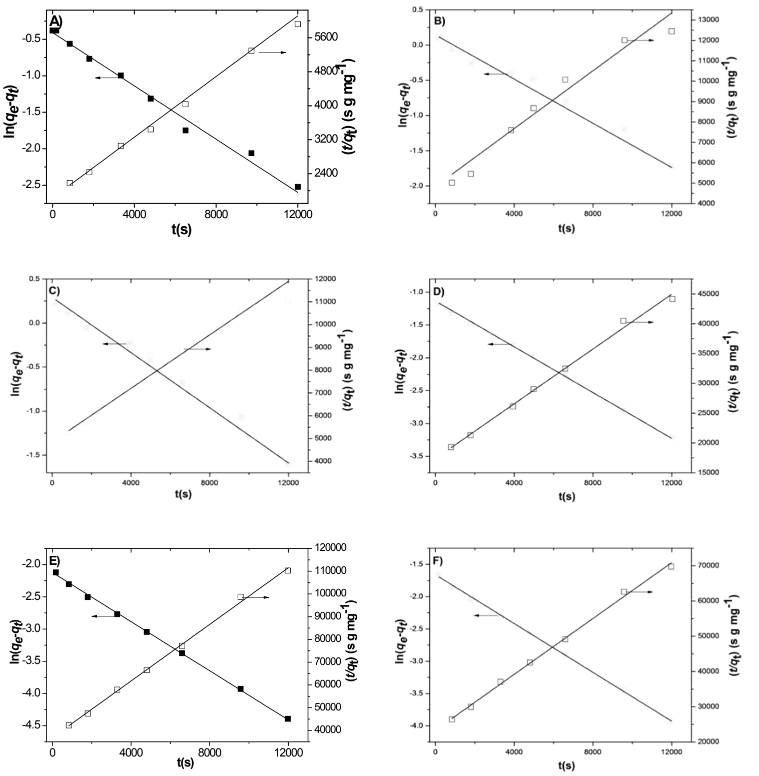

Figure S3. Representative plots of the fitting of linearized forms of pseudo-first (■) and pseudo-second (□) order equations to experimental sorption data for A) Ben (B) Tol (C)Xyl(D) Pyr (E) (B(b)F and (F) B(a)P onto the Pec-PVA/CS hydrogel, at 25ºC.

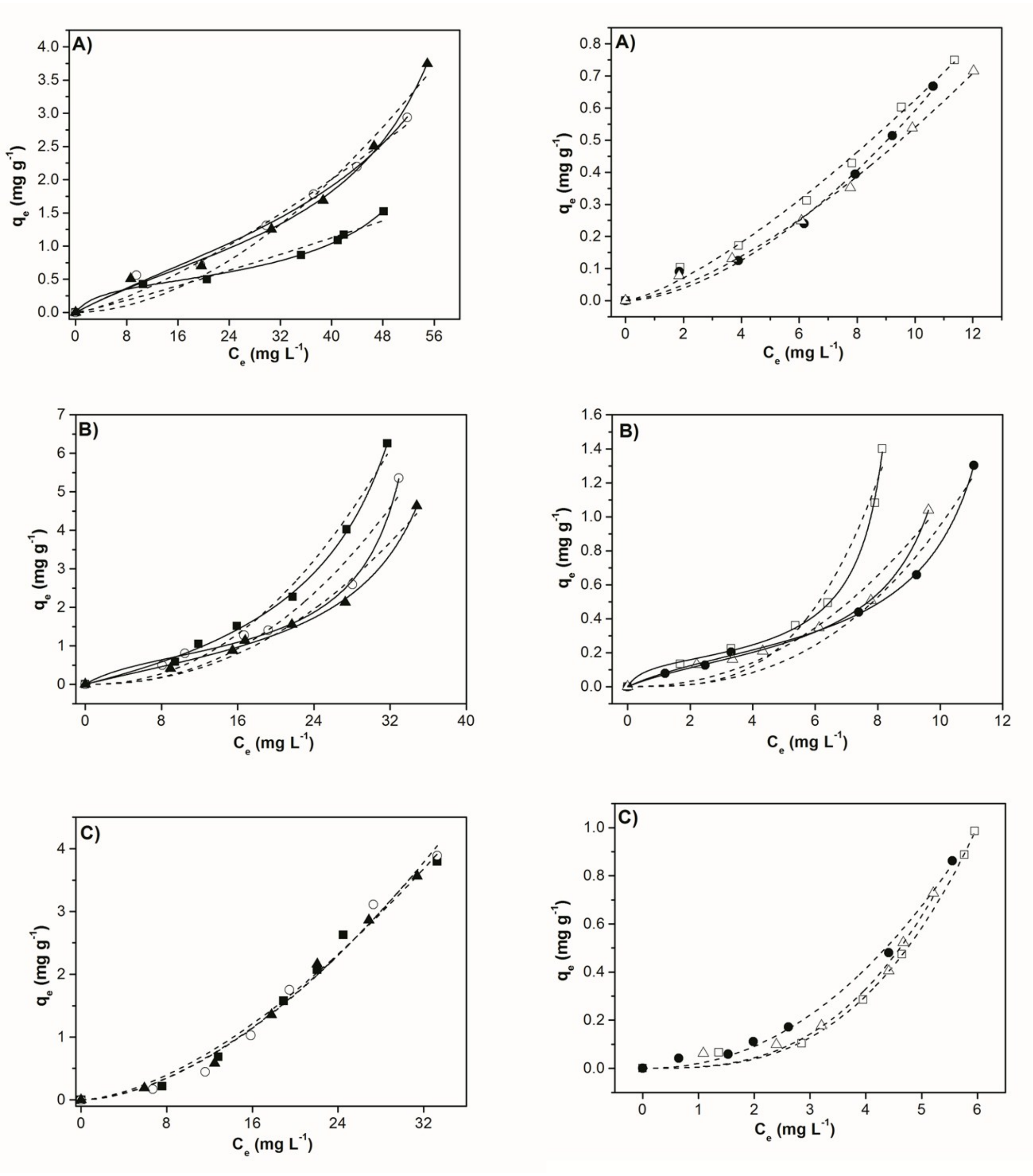

Figure S4. Representative sorption isotherms of benzene, toluene, xylenes, pyrene, B(b)F and B(a)P, from aqueous solutions, by the (A) Pec/CS, (B) Pec-β-CD/CS and (C) Pec-PVA/CS hydrogels, at 25ºC. Solid and dashed lines correspond to the fitting of BET and Freundlich models to the experimental data, respectively. A) to C) Ben (■), Tol (o), Xyl (▲), Pyr (⭘), B(b)F (●) and B(a)P (❐).

Table S1. Typical concentrations of BTXs and PAHs in petroleum and petroleum derivatives, and contaminated environmental media.

Hydrocarbon	Source	concentration (mg/L)	ref.
Benzene	Soil sample, two months after an oil spill (Nigeria).	73.2 (±14.0)	1
	Crude oil	1870 (±20)	2
	Sea water	0.0008	3,4
	Gasoline	900 (±100)	5
Toluene	Soil sample, two months after an oil spill (Nigeria).	19.2 (±12.5)	1
	Crude oil	6610 (±30)	2
	Gasoline	7500 (±100)	5
Xylenes	Soil sample, two months after an oil spill (Nigeria).	21.7 (±4.6)	1
	Crude oil	3000	2
	Gasoline	13100 (±400)	5
Pyrene	Crude oil	9.2	6
	Gasoline	110(±10)	5
	Produced water in oil production offshore and onshore (PW)*	6.30×10^{-4}	7,8
B(b)F	Crude oil	14	6
	Gasoline	4(±0.3)	5
	PW	2.80×10^{-5}	7,8
B(a)P	Crude oil	7.7	6
	Gasoline	50(±0.2)	5
	PW	5.20×10^{-4}	7,8

*PW - generally consists of a mixture of formation water contained naturally in the reservoir, injected water user for the recovery of oil and treatment chemicals added during production[6,7]

Table S2. Composition of the BTXs and PHAs mixed solutions used to obtain the sorption isotherms, at 25 ºC, for the indicated adsorbents.

	C_0 (mg L^{-1})					
Pec/CS	Benzene	Toluene	Xylenes	Pyrene	B(b)F	B(a)P
C1S	11.5(±0.2)*	10.5(±0.3)	9.5(±0.2)	2.1(±0.1)	2.0(±0.1)	2.0(±0.1)
C2S	22.5(±0.7)	21.5(±0.5)	21.6(±0.5)	4.3(±0.1)	4.2(±0.1)	4.0(±0.1)
C3S	38.7(±1.0)	32.9(±0.9)	33.7(±0.8)	6.9(±0.2)	6.7(±0.2)	6.7(±0.2)
C4S	44.9(±1.1)	41.09(±1.03)	42.7(±1.1)	8.7(±0.2)	8.6(±0.2)	8.6(±0.2)
C5S	45.2(±1.1)	48.4(±1.2)	51.3(±1.3)	10.5(±0.3)	9.7(±0.2)	10.8(±0.3)
C6S	53.1(±1.3)	56.9(±1.4)	60.5(±1.5)	12.5(±0.3)	11.5(±0.3)	13.1(±0.8)
Pec-β-CD/CS						
B1S	11.4(±0.2)	9.6(±0.2)	10.5(±0.2)	2.1(±0.1)	1.47(±0.04)	2.7(±0.1)
B2S	14.5(±0.3)	12.5(±0.2)	18.4(±0.4)	4.1(±0.1)	3.0(±0.1)	4.0(±0.1)
B3S	19.3(±0.4)	19.9(±0.4)	19.7(±0.4)	6.7±0.2)	4.0(±0.1)	5.1(±0.1)
B4S	26.9(±0.5)	23.0(±0.5)	25.5(±0.5)	8.1(±0.2)	8.9(±0.3)	7.3(±0.1)
B5S	33.3(±0.7)	33.4(±0.7)	32.1(±0.6)	9.9(±0.3)	11.2(±0.3)	9.3(±0.2)
B6S	38.7(±0.8)	38.9(±0.8)	41.0(±0.8)	10.1(±0.3)	13.5(±0.4)	11.5(±0.2)
Pec-PVA/CS						
P1S	8.8(±0.2)	7.7(±0.2)	7.0(±0.2)	1.63(±0.04)	0.76(±0.02)	1.25(±0.04)
P2S	15.1(±0.3)	13.1(±0.3)	14.7(±0.4)	3.4(±0.1)	1.79(±0.04)	2.7(±0.1)
P3S	21.5(±0.4)	17.9(±0.4)	21.2(±0.6)	4.8(±0.1)	2.3(±0.1)	3.7(±0.1)
P4S	25.7(±0.5)	22.1(±0.4)	25.9(±0.8)	5.6(±0.1)	3.1(±0.1)	5.1(±0.2)
P5S	28.4(±0.6)	31.1(±0.6)	31.5(±0.9)	7.0(±0.2)	5.3(±0.1)	5.5(±0.2)
P6S	38.6(±0.8)	38.1(±0.8)	37.1(±1.1)	7.2(±0.2)	6.6(±0.2)	6.1(±0.2)

*values inside brackets are standard deviations of the average

Table S3. Composition of mixed BTXs and PHAs solutions used to measure sorption kinetics onto the indicated hydrogels, at 25 ºC.

	C_0 (mg L^{-1})					
Pec/CS	Benzene	Toluene	Xylenes	Pyrene	B(b)F	B(a)P
C1K	11.5(±0.2)*	10.5(±0.2)	9.5(±0.2)	2.10(±0.04)	2.01(±0.03)	2.01(±0.04)
C2K	22.5(±0.4)	21.5(±0.4)	21.6(±0.4)	4.3(±0.1)	4.2(±0.1)	4.0(±0.1)
C3K	38.7(±0.8)	32.9(±0.5)	33.7(±0.7)	6.9(±0.1)	6.7(±0.1)	6.7(±0.1)
C4K	44.9(±0.9)	41.1(±0.8)	51 (±1)	8.6(±0.2)	8.6(±0.1)	10.8(±0.2)
C5K	53 (±1)	57 (±1)	60 (±1)	12.5(±0.3)	11.5(±0.2)	13.1(±0.3)
Pec-β-CD/CS						
B1K	11.4(±0.6)	9.8(±0.5)	10.3(±0.5)	2.1(±0.1)	1.5(±0.1)	2.7(±0.1)
B2K	15.9(±0.8)	13.5(±0.7)	18.8(±0.9)	4.2(±0.2)	3.0(±0.2)	5.1(±0.3)
B3K	27(±1)	23(±1)	24.4(±1.3)	7.3(±0.4)	4.4(±0.2)	7.3(±0.4)
B4K	34 (±2)	32(±2)	36(±2)	8.4(±0.4)	5.9(±0.3)	9.8(±0.5)
B5K	39(±2)	33(±2)	43(±2)	9.9(±0.5)	6.7(±0.3)	11.5(±0.6)
Pec-PVA/CS						
P1K	8.8(±0.4)	7.7(±0.4)	7.0(±0.4)	1.7(±0.1)	0.89(±0.04)	1.4(±0.1)
P2K	15.1(±0.8)	13.1(±0.7)	14.4(±0.7)	3.4(±0.2)	1.7(±0.1)	2.7(±0.1)
P3K	21 (±1)	18.9(±1)	21(±1)	4.8(±0.2)	2.3(±0.1)	3.7(±0.2)
P4K	23 (±1)	18.8(±0.9)	22(±1)	6.0(±0.3)	3.1(±0.2)	4.9(±0.2)
P5K	28 (±1)	25.3(±1)	31(±2)	7.1(±0.4)	3.4(±0.2)	5.5(±0.3)

*values inside brackets are standard deviations of the average

Table S4. Fitting kinetic parameters for the simultaneous sorption of BTXs and some PAHs onto the Pec/CS hydrogel, at 25 ºC.

	$q_{e,exp}$ (mg g^{-1})	$q_{e,1}$ (mg g^{-1})	k_1 (10^{-4} g mg^{-1} s^{-1})	*AIC*	$q_{e,2}$ (mg g^{-1})	k_2 (10^{-4} g mg^{-1} s^{-1})	*AIC*
**Benzene**							
C1K	0.42(±0.01)*	0.41(±0.02)	1.16(±0.02)	3.20	0.46(±0.04)	0.37(±0.03)	12.95
C2K	0.49(±0.01)	0.48(±0.02)	1.16(±0.02)	3.24	0.54(±0.04)	0.52(±0.04)	12.89
C3K	0.86(±0.03)	0.83(±0.02)	1.17(±0.02)	3.25	0.93(±0.02)	1.56(±0.04)	12.41
C4K	1.09(±0.03)	1.05(±0.02)	1.16(±0.02)	3.18	1.17(±0.02)	2.47(±0.04)	12.21
C5K	1.5(±0.1)	1.46(±0.02)	1.16(±0.02)	3.20	1.64(±0.02)	4.84(±0.04)	11.92
**Toluene**							
	$q_{e,exp}$ (mg g^{-1})	$q_{e,1}$ (mg g^{-1})	k_1 (10^{-4} g mg^{-1} s^{-1})	*AIC*	$q_{e,2}$ (mg g^{-1})	k_2 (10^{-4} g mg^{-1} s^{-1})	*AIC*
C1K	0.40(±0.01)*	0.39(±0.01)	1.16(±0.02)	3.25	0.44(±0.06)	0.35(±0.03)	13.11
C2K	0.71(±0.02)	0.69(±0.02)	1.16(±0.02)	4.79	0.77(±0.03)	1.07(±0.02)	13.65
C3K	1.31(±0.04)	1.26(±0.02)	1.16(±0.02)	4.39	1.41(±0.02)	3.60(±0.03)	13.12
C4K	1.8(±0.1)	1.71(±0.02)	1.16(±0.02)	3.25	1.92 (±0.04)	6.63(±0.04)	11.78
C5K	2.9(±0.1)	2.82(±0.03)	1.16(±0.03)	3.21	3.16(±0.02)	11.81(±0.03)	11.35
**Xylenes**							
	$q_{e,exp}$ (mg g^{-1})	$q_{e,1}$ (mg g^{-1})	k_1 (10^{-4} g mg^{-1} s^{-1})	*AIC*	$q_{e,2}$ (mg g^{-1})	k_2 (10^{-4} g mg^{-1} s^{-1})	*AIC*
C1K	0.37(±0.01)	0.35(±0.02)	1.16(±0.02)	5.66	0.4(±0.1)	0.28(±0.02)	13.15
C2K	0.70(±0.02)	0.67(±0.02)	1.16(±0.03)	4.84	0.75(±0.03)	1.02(±0.02)	12.59
C3K	1.25(±0.04)	1.20(±0.02)	1.16(±0.02)	4.22	1.34(±0.02)	3.28(±0.03)	12.09
C4K	2.5(±0.1)	2.4(±0.02)	1.16(±0.02)	3.18	2.69(±0.02)	13.1(±0.1)	11.48
C5K	3.7(±0.1)	3.6(±0.02)	1.17(±0.02)	3.15	4.03(±0.04)	29.4(±0.1)	11.14
**Pyrene**							
	$q_{e,exp}$ (mg g^{-1})	$q_{e,1}$ (mg g^{-1})	k_1 (10^{-4} g mg^{-1} s^{-1})	*AIC*	$q_{e,2}$ (mg g^{-1})	k_2 (10^{-5} g mg^{-1} s^{-1})	*AIC*
C1K	0.09(±0.01)	0.09(±0.02)	1.16(±0.02)	3.22	0.09(±0.02)	0.17(±0.02)	14.37
C2K	0.17(±0.01)	0.16(±0.02)	1.16(±0.02)	3.15	0.2(±0.1)	0.62(±0.02)	13.81
C3K	0.31(±0.01)	0.30(±0.02)	1.16(±0.02)	3.25	0.3(±0.1)	2.04(±0.01)	13.29
C4K	0.43(±0.01)	0.41(±0.02)	1.15(±0.01)	3.25	0.46(±0.02)	3.8(±0.02)	13.02

C5K	0.75(±0.02)	0.72(±0.02)	1.16(±0.02)	3.25	0.81(±0.01)	11.8(±0.1)	12.53
				B(b)F			
	$q_{e,exp}$ (mg g^{-1})	$q_{e,1}$ (mg g^{-1})	k_1 (10^{-4} g mg^{-1} s^{-1})	*AIC*	$q_{e,2}$ (mg g^{-1})	k_2 (10^{-5} g mg^{-1} s^{-1})	*AIC*
C1K	0.06(±0.01)	0.06(±0.02)	1.16(±0.01)	3.24	0.07(±0.02)	0.09(±0.01)	14.65
C2K	0.12(±0.02)	0.12(±0.01)	1.16(±0.01)	3.25	0.13(±0.02)	0.33(±0.03)	14.09
C3K	0.24(±0.02)	0.23(±0.02)	1.15(±0.02)	3.24	0.26(±0.03)	1.2(±0.1)	13.52
C4K	0.39(±0.01)	0.38(±0.02)	1.16(±0.02)	3.25	0.4(±0.1)	3.3(±0.1)	13.09
C5K	0.67(±0.02)	0.64(±0.02)	1.16(±0.01)	3.23	0.72(±0.03)	9.33(±0.1)	12.63
				B(a)P			
	$q_{e,exp}$ (mg g^{-1})	$q_{e,1}$ (mg g^{-1})	k_1 (10^{-4} g mg^{-1} s^{-1})	*AIC*	$q_{e,2}$ (mg g^{-1})	k_2 (10^{-5} g mg^{-1} s^{-1})	*AIC*
C1K	0.07(±0.01)	0.07(±0.02)	1.16(±0.02)	3.23	0.08(±0.01)	0.10(±0.02)	14.59
C2K	0.13(±0.02)	0.13(±0.02)	1.16(±0.02)	3.25	0.14(±0.02)	0.36(±0.02)	14.05
C3K	0.24(±0.02)	0.24(±0.02)	1.16(±0.01)	3.24	0.27(±0.01)	1.30(±0.01)	13.49
C4K	0.54(±0.01)	0.52(±0.02)	1.16(±0.01)	3.25	0.57(±0.04)	6.05(±0.01)	12.82
C5K	0.72(±0.02)	0.69(±0.02)	1.16(±0.02)	3.23	0.77(±0.03)	10.7(±0.1)	12.57

*values inside brackets are standard deviations of the average

Table S5. Fitting kinetic parameters for the simultaneous sorption of BTXs and some PAHs onto the Pec-β-CD/CShydrogel, at 25 ºC.

			Benzene				
	$q_{e,exp}$ (mg g^{-1})	$q_{e,1}$ (mg g^{-1})	k_1 (10^{-4} g mg^{-1} s^{-1})	*AIC*	$q_{e,2}$ (mg g^{-1})	k_2 (10^{-3} g mg^{-1} s^{-1})	*AIC*
B1K	0.58(±0.02)	0.61(±0.01)	1.95(±0.05)	4.16	0.62(±0.04)	0.09(±0.01)	12.66
B2K	1.08(±0.03)	1.2(±0.1)	1.76(±0.05)	3.89	1.17(±0.02)	0.26(±0.01)	12.05
B3K	2.3(±0.1)	2.37(±0.03)	1.71(±0.05)	3.44	2.45(±0.01)	1.20(±0.01)	11.40
B4k	2.8(±0.1)	2.87(±0.04)	1.72(±0.04)	3.63	3.01(±0.01)	2.08(±0.01)	10.88
B5K	4.02(±0.12)	4.3(±0.1)	1.87(±0.05)	3.85	4.33(±0.01)	4.01(±0.01)	10.88
			Toluene				
	$q_{e,exp}$ (mg g^{-1})	$q_{e,1}$ (mg g^{-1})	k_1 (10^{-4} g mg^{-1} s^{-1})	*AIC*	$q_{e,2}$ (mg g^{-1})	k_2 (10^{-3} g mg^{-1} s^{-1})	*AIC*
B1K	0.49(±0.01)	0.5(±0.1)	1.95(±0.01)	4.18	0.54(±0.04)	0.06(±0.04)	12.74
B2K	0.83(±0.02)	0.9(±0.1)	1.63(±0.01)	4.10	0.90(±0.02)	0.15(±0.04)	12.25
B3K	1.7(±0.1)	1.9(±0.1)	2.28(±0.01)	3.99	1.80(±0.02)	0.91(±0.04)	11.87
B4K	2.4(±0.1)	2.1(±0.1)	1.93(±0.02)	4.59	2.45(±0.01)	1.42(±0.04)	10.69
B5K	2.6(±0.1)	2.8(±0.1)	1.54(±0.01)	4.21	2.81(±0.01)	2.45(±0.04)	11.51
			Xylenes				
	$q_{e,exp}$ (mg g^{-1})	$q_{e,1}$ (mg g^{-1})	k_1 (10^{-4} g mg^{-1} s^{-1})	*AIC*	$q_{e,2}$ (mg g^{-1})	k_2 (10^{-3} g mg^{-1} s^{-1})	*AIC*
B1K	0.41(±0.01)	0.4(±0.1)	1.59(±0.01)	4.05	0.44(±0.04)	0.05(±0.04)	12.58
B2K	0.90(±0.03)	0.95(±0.04)	1.62(±0.01)	4.08	0.98(±0.02)	0.18(±0.04)	12.19
B3K	1.7(±0.1)	1.9(±0.1)	2.07(±0.02)	4.27	1.86(±0.02)	0.84(±0.04)	12.02
B4K	3.3(±0.1)	3.4(±0.1)	1.6(±0.1)	3.57	3.59(±0.01)	2.53(±0.04)	10.73
B5K	3.8(±0.1)	3.93(±0.03)	1.74(±0.04)	4.18	4.09(±0.01)	3.78(±0.04)	11.20
			Pyrene				
	$q_{e,exp}$ (mg g^{-1})	$q_{e,1}$ (mg g^{-1})	k_1 (10^{-4} g mg^{-1} s^{-1})	*AIC*	$q_{e,2}$ (mg g^{-1})	k_2 (10^{-4} g mg^{-1} s^{-1})	*AIC*
B1K	0.13(±0.01)	0.14(±0.04)	1.7(±0.1)	3.87	0.14(±0.01)	0.04(±0.01)	14.03
B2K	0.21(±0.01)	0.22(±0.01)	1.68(±0.01)	4.20	0.22(±0.01)	0.09(±0.04)	13.50
B3K	0.49(±0.01)	0.53(±0.05)	1.80(±0.01)	4.04	0.53(±0.04)	0.55(±0.04)	12.78
B4K	0.88(±0.02)	0.94(±0.06)	1.71(±0.01)	4.19	0.96(±0.03)	1.69(±0.04)	12.54

B5K	2.08(±0.03)	2.12(±0.05)	1.8(±0.1)	3.99	2.13(±0.01)	9.13(±0.04)	12.03
B(b)F							
	$q_{e,exp}$ (mg g^{-1})	$q_{e,1}$ (mg g^{-1})	k_1 (10^{-4} g mg^{-1} s^{-1})	*AIC*	$q_{e,2}$ (mg g^{-1})	k_2 (10^{-5} g mg^{-1} s^{-1})	*AIC*
B1K	0.08(±0.02)	0.08(±0.04)	1.79(±0.04)	3.72	0.08(±0.01)	0.16(±0.01)	14.19
B2K	0.13(±0.01)	0.14(±0.04)	1.92(±0.04)	3.89	0.14(±0.01)	0.46(±0.01)	13.76
B3K	0.32(±0.01)	0.33(±0.04)	1.91(±0.04)	3.94	0.34(±0.04)	2.71(±0.04)	12.94
B4K	0.59(±0.01)	0.63(±0.04)	1.93(±0.03)	3.83	0.63(±0.01)	9.54(±0.01)	12.43
B5K	0.65(±0.02)	0.69(±0.01)	1.91(±0.01)	3.89	0.69(±0.02)	11.6(±0.01)	12.31
B(a)P							
	$q_{e,exp}$ (mg g^{-1})	$q_{e,1}$ (mg g^{-1})	k_1 (10^{-4} g mg^{-1} s^{-1})	*AIC*	$q_{e,2}$ (mg g^{-1})	k_2 (10^{-4} g mg^{-1} s^{-1})	*AIC*
B1K	0.13(±0.01)	0.13(±0.04)	1.73(±0.01)	4.02	0.14(±0.01)	0.06(±0.04)	13.89
B2K	0.22(±0.01)	0.23(±0.04)	1.92(±0.01)	3.89	0.23(±0.01)	0.13(±0.01)	13.34
B3K	0.50(±0.02)	0.53(±0.04)	1.91(±0.01)	3.87	0.53(±0.03)	0.71(±0.04)	12.53
B4K	0.91(±0.03)	0.96(±0.04)	1.93(±0.01)	3.75	0.97(±0.02)	2.33(±0.04)	12.05
B5K	1.04(±0.03)	1.10(±0.04)	1.9(±0.1)	3.82	1.11(±0.02)	2.98(±0.01)	11.94

*values inside brackets are standard deviations of the average

Table S6. Fitting kinetic parameters for the simultaneous sorption of BTXs and some PAHs onto the Pec-PVA/CShydrogel, at 25 ºC.

Benzene							
	$q_{e,exp}$ (mg g^{-1})	$q_{e,1}$ (mg g^{-1})	k_1 (10^{-4} g mg^{-1} s^{-1})	*AIC*	$q_{e,2}$ (mg g^{-1})	k_2 (10^{-4} g mg^{-1} s^{-1})	*AIC*
P1K	0.21(±0.01)	0.22(±0.02)	1.68(±0.05)	3.46	0.22(±0.02)	0.02 (±0.01)	13.23
P2K	0.68(±0.02)	0.7(±0.1)	1.68(±0.05)	3.73	0.69 (±0.01)	0.15(±0.01)	9.54
P3K	1.07(±0.03)	1.04(±0.08)	1.38(±0.05)	4.10	1.09(±0.02)	0.26(±0.01)	12.10
P4K	1.7(±0.1)	1.8(±0.1)	1.56(±0.04)	4.27	1.74(±0.02)	0.56(±0.01)	12.11
P5K	2.6(±0.1)	2.6(±0.1)	1.62(±0.05)	3.96	2.67(±0.02)	1.97(±0.02)	12.19
Toluene							
	$q_{e,exp}$ (mg g^{-1})	$q_{e,1}$ (mg g^{-1})	k_1 (10^{-4} g mg^{-1} s^{-1})	*AIC*	$q_{e,2}$ (mg g^{-1})	k_2 (10^{-4} g mg^{-1} s^{-1})	*AIC*
P1K	0.17(±0.01)	0.2(±0.1)	1.59(±0.01)	4.24	0.17(±0.02)	0.92(±0.01)	13.19
P2K	0.44(±0.01)	0.4(±0.1)	1.94(±0.02)	3.99	0.45(±0.01)	0.85(±0.02)	12.80
P3K	1.10(±0.03)	1.2(±0.1)	1.56(±0.01)	4.20	1.13(±0.02)	2.61(±0.02)	12.31
P4K	1.29(±0.04)	1.4(±0.1)	1.41(±0.01)	4.12	1.32(±0.02)	2.73(±0.02)	12.39
P5K	2.4(±0.1)	2.7(±0.1)	1.39(±0.01)	4.22	2.52(±0.02)	7.82(±0.02)	12.32
Xylenes							
	$q_{e,exp}$ (mg g^{-1})	$q_{e,1}$ (mg g^{-1})	k_1 (10^{-4} g mg^{-1} s^{-1})	*AIC*	$q_{e,2}$ (mg g^{-1})	k_2 (10^{-4} g mg^{-1} s^{-1})	*AIC*
P1K	0.19(±0.01)	0.21(±0.04)	1.61(±0.02)	3.81	0.2(±0.1)	0.06(±0.01)	14.13
P2K	0.58(±0.02)	0.6(±0.1)	2.2(±0.1)	4.48	0.59(±0.01)	1.59(±0.02)	12.50
P3K	1.25(±0.04)	1.3(±0.1)	1.6(±0.1)	4.16	1.27(±0.02)	3.3(±0.1)	12.20
P4K	1.54(±0.04)	1.6(±0.1)	1.39(±0.01)	4.25	1.58(±0.01)	4.6 (±0.1)	12.11
P5K	2.9(±0.1)	2.5(±0.1)	1.73(±0.02)	3.81	2.89(±0.03)	5.1(±0.1)	10.83
Pyrene							
	$q_{e,exp}$ (mg g^{-1})	$q_{e,1}$ (mg g^{-1})	k_1 (10^{-4} g mg^{-1} s^{-1})	*AIC*	$q_{e,2}$ (mg g^{-1})	k_2 (10^{-4} g mg^{-1} s^{-1})	*AIC*
P1K	0.07(±0.01)	0.07(±0.04)	1.7(±0.1)	3.93	0.07(±0.01)	0.01(±0.01)	14.72
P2K	0.16(±0.01)	0.17(±0.04)	1.8(±0.1)	3.80	0.17(±0.03)	0.07(±0.01)	13.60
P3K	0.30(±0.01)	0.31(±0.03)	1.7(±0.1)	3.70	0.31(±0.01)	0.25(±0.01)	13.07
P4K	0.47(±0.01)	0.48(±0.04)	1.7(±0.1)	3.78	0.48(±0.01)	0.62(±0.01)	12.64

P5K	0.89(±0.02)	0.91(±0.04)	1.7(±0.1)	3.73	0.90(±0.01)	2.02(±0.01)	12.24
B(b)F							
	$q_{e,exp}$ (mg g^{-1})	$q_{e,1}$ (mg g^{-1})	k_1 (10^{-4} g mg^{-1} s^{-1})	*AIC*	$q_{e,2}$ (mg g^{-1})	k_2 (10^{-5} g mg^{-1} s^{-1})	*AIC*
P1K	0.04(±0.01)	0.04(±0.01)	1.6(±0.1)	3.88	0.04(±0.01)	0.04(±0.01)	15.16
P2K	0.06(±0.01)	0.06(±0.03)	1.9(±0.1)	3.70	0.06(±0.04)	0.12(±0.01)	14.31
P3K	0.11(±0.01)	0.12(±0.03)	1.9(±0.1)	3.65	0.12(±0.03)	0.47(±0.01)	13.78
P4K	0.17(±0.01)	0.17(±0.04)	1.9(±0.1)	3.75	0.17(±0.02)	1.1(±0.1)	13.19
P5K	0.33(±0.01)	0.33(±0.04)	1.9(±0.1)	3.64	0.34(±0.01)	3.59(±0.01)	12.84
B(a)P							
	$q_{e,exp}$ (mg g^{-1})	$q_{e,1}$ (mg g^{-1})	k_1 (10^{-4} g mg^{-1} s^{-1})	*AIC*	$q_{e,2}$ (mg g^{-1})	k_2 (10^{-5} g mg^{-1} s^{-1})	*AIC*
P1K	0.06(±0.01)	0.07(±0.04)	1.7(±0.1)	3.87	0.06(±0.04)	0.08(±0.01)	14.83
P2K	0.09(±0.02)	0.10(±0.04)	1.9(±0.1)	3.73	0.10(±0.04)	0.3(±0.1)	13.87
P3K	0.19(±0.01)	0.19(±0.04)	1.9(±0.1)	3.70	0.19(±0.02)	1.17(±0.04)	13.37
P4K	0.26(±0.01)	0.26(±0.04)	1.9(±0.1)	3.81	0.27(±0.01)	2.47(±0.04)	12.83
P5K	0.52(±0.02)	0.52(±0.03)	1.9(±0.1)	3.66	0.53(±0.01)	9.12(±0.04)	12.42

*values inside brackets are standard deviations of the average

References

1 I. Osuji and O. Achugasim, *J. Appl. Sci. Environ. Manag.*, 2010, **14**, 17–20.

2 P. L. Grizzle and H. J. Coleman, *Anal. Chem.*, 1979, **51**, 602–608.

3 IPCS/WHO, *Benzene.Environmental Health Criteria 150*, . International Programme on Chemical Safety, World Health Organization., Geneva, Switzerland, 1993.

4 G. Pezzagno, L. Maestri and M. L. Fiorentino, *Am. J. Ind. Med.*, 1999, **35**, 511–518.

5 C. M. C. Filho, T. Matias, L. Durães and A. J. M. Valente, *Colloids Surfaces A Physicochem. Eng. Asp.*, 2017, **520**, 550–560.

6 D. M. Pampanin and M. O. Sydnes, in *Hydrocarbon*, eds. V. . Kutcherov and A. Kolesnikov, InTech, 2013, p. 216.

7 O. G. P. P. W. Fate and E. T. Force, *Fate and Effects of Naturally Occurring Substances in Produced Water on the Marine Environment*, Technical Report, 2005.

8 T. I. Røe Utvik, *Chemosphere*, 1999, **39**, 2593–2606.
